# Rational Design of Antiangiogenic Helical Oligopeptides Targeting the Vascular Endothelial Growth Factor Receptors

**DOI:** 10.3389/fchem.2019.00170

**Published:** 2019-03-29

**Authors:** Simone Zanella, Gianfranco Bocchinfuso, Marta De Zotti, Daniela Arosio, Franca Marino, Stefano Raniolo, Luca Pignataro, Giovanni Sacco, Antonio Palleschi, Alvaro S. Siano, Umberto Piarulli, Laura Belvisi, Fernando Formaggio, Cesare Gennari, Lorenzo Stella

**Affiliations:** ^1^Department of Chemistry, University of Milan, Milan, Italy; ^2^Department of Chemical Science and Technologies, University of Rome Tor Vergata, Rome, Italy; ^3^Padova Unit, Department of Chemistry, Institute of Biomolecular Chemistry, CNR, University of Padova, Padova, Italy; ^4^National Research Council, Institute of Molecular Science and Technologies, Milan, Italy; ^5^Center for Research in Medical Pharmacology, University of Insubria, Varese, Italy; ^6^Departamento de Química Organica, Facultad de Bioquímica y Ciencias Biologicas, Universidad Nacional del Litoral, Santa Fe, Argentina

**Keywords:** helical folded peptides, protein-protein interactions, C^α,α^-disubstituted amino acids, VEGF-C, angiogenesis

## Abstract

Tumor angiogenesis, essential for cancer development, is regulated mainly by vascular endothelial growth factors (VEGFs) and their receptors (VEGFRs), which are overexpressed in cancer cells. Therefore, the VEGF/VEGFR interaction represents a promising pharmaceutical target to fight cancer progression. The VEGF surface interacting with VEGFRs comprises a short α-helix. In this work, helical oligopeptides mimicking the VEGF-C helix were rationally designed based on structural analyses and computational studies. The helical conformation was stabilized by optimizing intramolecular interactions and by introducing helix-inducing C^α,α^-disubstituted amino acids. The conformational features of the synthetic peptides were characterized by circular dichroism and nuclear magnetic resonance, and their receptor binding properties and antiangiogenic activity were determined. The best hits exhibited antiangiogenic activity *in vitro* at nanomolar concentrations and were resistant to proteolytic degradation.

## Introduction

Angiogenesis—i.e., the formation of new blood vasculature from the established blood vessel network—can be associated to both physiological and pathological processes (e.g., inflammation, tumor growth, and metastasis). Among the latter, tumor angiogenesis is essential for cancer development, since neovascularization provides a steady supply of oxygen and nutrients, supporting the proliferation of cancer cells (Mizejewski, 1999; Danhier et al., 2012; Johannessen et al., 2013). For this reason, antiangiogenic agents are used to impede or retard cancer progression and metastasis (Ferrara and Adamis, 2016).

Different receptors are involved in angiogenesis regulation, and their expression depends on the conditions of the cell environment (e.g., pH, oxygen or supply of nutrients) (Mizejewski, 1999). Angiogenic processes are also mediated by cross-talk mechanisms that trigger direct association and cluster formation between specific receptors. For example, the cooperation between integrins and vascular endothelial growth factor receptors (VEGFRs) was found to be crucial in pathological processes such as tumor growth and development (Somanath et al., 2009; Desgrosellier and Cheresh, 2010).

VEGFRs are receptor tyrosine kinases (RTKs) that have a central role in tumor angiogenesis and progression. Indeed, the hypoxic conditions characteristic of the tumor environment induce both up-regulation of VEGFRs and gene expression of vascular endothelial growth factors (VEGFs) (Ferrara et al., 2003; Hoeben et al., 2004; Olsson et al., 2006; Carmeliet and Jain, 2011; Shibuya, 2013). In addition to the tumor promoting effects of neovascularization, autocrine VEGF/VEGFR signaling favors growth, proliferation and migration of cancer cells (Su et al., 2007; Simon et al., 2017).

Dimerization and activation of VEGFRs are triggered by binding of VEGFs to the extracellular domain of the receptors (Ferrara and Adamis, 2016). The VEGF family consists of five members [VEGF-A, VEGF-B, VEGF-C, VEGF-D, and PlGF (placental growth factor)], and there are three distinct receptors (VEGFR-1, VEGFR-2, VEGFR-3). The various growth factors have differential selectivity: for instance, VEGF-A binds to VEGFR-1 and VEGFR-2, while VEGF-C binds to VEGFR-2 and VEGFR-3. In addition, the three receptors have different functions, with VEGFR-2 being mainly involved in angiogenesis and VEGFR-3 regulating lymphangiogenesis (Ferrara and Adamis, 2016; Nasir, 2019).

Three main approaches targeting VEGF-A/VEGFR-2 signaling in human cancer have been approved for clinical practice (Nasir, 2019). One strategy involves inhibition of the tyrosine kinase activity of VEGFR-2 by small molecules interacting with the intracellular segment of the receptor, such as sorafenib and sunitinib (Musumeci et al., 2012). Alternatively, VEGF-mediated angiogenesis can be impaired by blocking the VEGF-A/VEGFR-2 interaction. This has been accomplished by binding and neutralizing circulating VEGF-A with monoclonal antibodies (bevacizumab) or recombinant proteins mimicking the receptor (aflibercept) (Ferrara et al., 2004; Ferrara and Adamis, 2016). Alternatively, VEGFR-2 has been targeted with therapeutic monoclonal antibodies, such as ramucirumab. These molecules are now a standard of care for the treatment of several metastatic cancers (Ferrara and Adamis, 2016), even though clinical results have not met in all cancer types the initial high hopes for this therapeutic strategy, underlining the need for more effective antiangiogenic drugs (Vasudev and Reynolds, 2014; Ronca et al., 2017). Anti-VEGF-A/VEGFR-2 therapy has proven more effective in ophthalmology, in the treatment of intraocular neo vascular disorders, such as age-related macular degeneration (Ferrara and Adamis, 2016). However, each of the above mentioned approved drugs has its own limitations: kinase inhibitors have limited selectivity, while antibodies suffer from poor pharmacokinetics, limited tissue penetration and high costs (Chames et al., 2009; Howard et al., 2015). Their use in the therapy of eye diseases requires repeated intravitreal injections, and new drugs that can be administered by simpler and safer routes are highly desirable (Sidman et al., 2015).

Peptides and peptidomimetics with a well-defined conformation (foldamers) represent a promising alternative to biological therapeutics for the inhibition of protein-protein interactions, and are currently experiencing a revival of interest from the pharmaceutical industry (Henninot et al., 2018). Among those able to interact with the extracellular domain of VEGFRs, it is worth mentioning the peptoid ligands described by Kodadek and colleagues (Udugamasooriya et al., 2008), the helical peptides developed starting from VEGF-A and Vammin hotspots (García-Aranda et al., 2013), and cyclopeptides isolated by phage display technique (Zilberberg et al., 2003), or rationally designed (Gautier et al., 2010). In 2011, D'Andrea and coworkers developed a α-helical decapentapeptide based on the natural sequence of VEGF-A, with potent inhibitory activity against VEGF-stimulated angiogenesis *in vivo* (Basile et al., 2011; Diana et al., 2013). Because of its synthetic accessibility and antiangiogenic properties, recently some of us selected that peptide to prepare a dual-action compound able to interfere with the integrin α_V_β_3_-VEGFR-1 cross-talk (Zanella et al., 2015). This conjugate was able to bind *in vitro* both integrin α_V_β_3_ and VEGFR-1, and exerted a strong antiangiogenic effect in VEGF-stimulated morphogenesis assays on human umbilical vein endothelial cells (HUVECs).

Herein, we report the results of our efforts to develop new VEGFR antagonists based on a helical fragment of VEGF-C, with promising activity against VEGF-mediated angiogenesis.

## Results

### Design

#### A Short Helix Is an Important Element of the VEGF/VEGFR Interface

**Figure 1:**
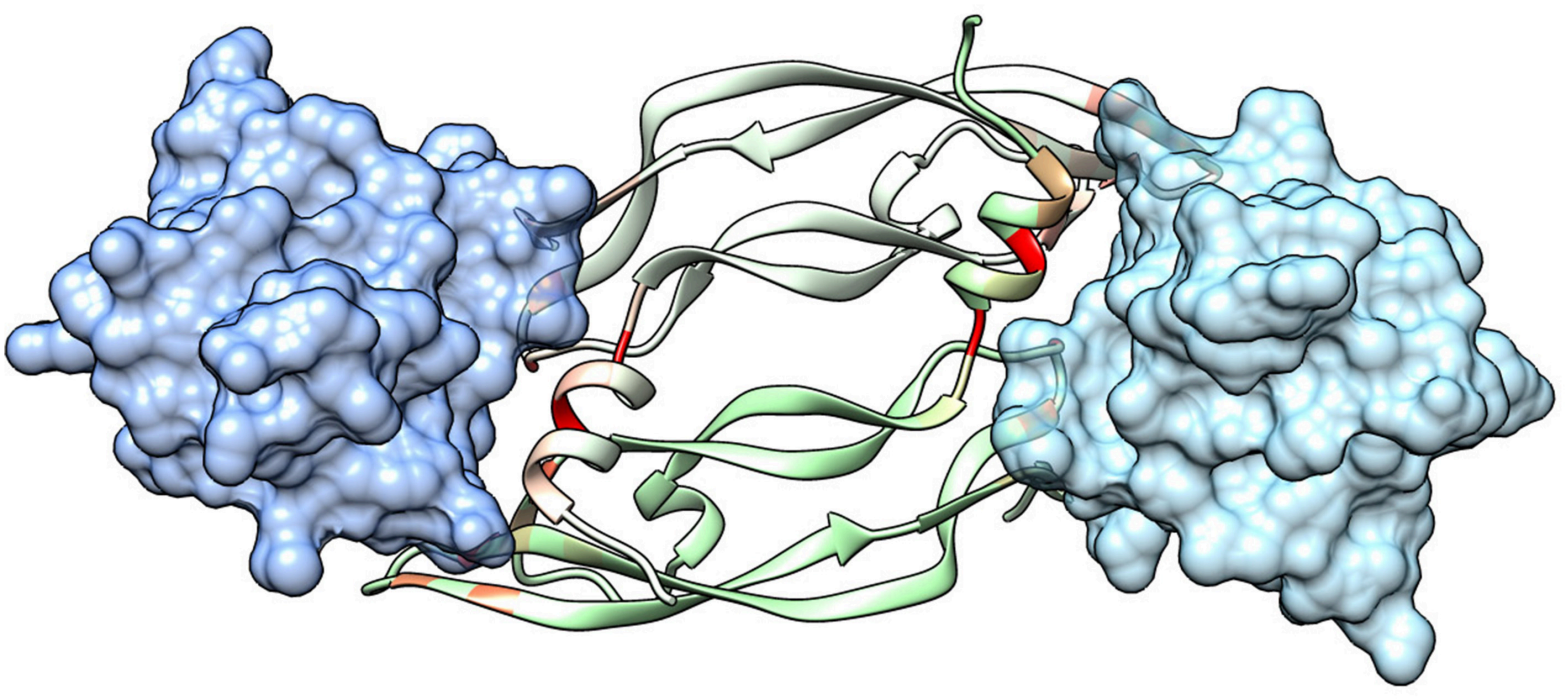
Structure of a VEGF/VEGFR complex (PDB code 1FLT). Ig-homology domain 2 of the receptor (VEGFR-1) is shown in surface representation (light blue and blue for the two subunits), while the two subunits of VEGF-A are shown as ribbons of two different shades of green. VEGF residues are colored depending on the standard binding free energy penalty associated with their mutation to Ala, as predicted by DrugScorePPI (values from 0 to 1.5 kcal/mol are reported in shades of color going from green to red; values 1.5 kcal/mol and above, up to the maximum of 2.4 kcal/mol, are all reported in red).

The extracellular domain of VEGFRs consists of seven Ig homology domains. VEGF binding takes place mostly on domain D2. Figure 1 shows the structure of the VEGF-A/VEGFR-1 (D2 domain) interface, whose main features are conserved in all VEGF/VEGFR complexes (Leppänen et al., 2013). We started our analysis from this complex, because all currently approved antiangiogenic drugs are targeted to VEGF-A or to VEGFR-1. VEGF residues are colored from green to red in order of increasing penalty in the standard binding free energy caused by their substitution to Ala, as predicted by *in silico* alanine scanning. This analysis indicates that a short helix, located at the N-terminal region of the growth factor, represents a significant portion of the interaction interface. Previous experimental studies have shown that this helix is involved in receptor specificity and protein dimerization of VEGFs (Siemeister et al., 1998; Robinson and Stringer, 2001; Leppänen et al., 2010). In the case of VEGF-A, the helix comprises residues 17–25 (a nonapeptide). Table 1 reports the numerical results of the *in silico* Ala scan for those residues, showing that the interaction with the receptor is mediated principally by amino acid residues at positions 1, 2, 5, 6, and 9 of the helical nonapeptide. This finding is consistent with experimental Ala scan data (Muller et al., 1997; Li et al., 2000). A similar analysis performed on the structure of the VEGF-A/VEGFR-2 complex confirmed the N-terminal helix as an important interacting element (Figure S1).

**Table 1:** *In silico* Ala scan of the VEGF-A helix interacting with VEGFR-1 (PDB code 1FLT)^a^.

**Position in the helix**	**Residue**	**FoldX**	**DrugScorePPI**	**KFC2**	**PCRPi**
		**$\Delta \Delta G_{\text{ binding}}^{{}^{\circ}}$ (kcal/mol)**	**$\Delta \Delta G_{\text{ binding}}^{{}^{\circ}}$ (kcal/mol)**	**Hot spots**	**Probability (%)**
1	Phe 17	1.6	0.4	X	48
2	Met 18	2.0	0.4	X	8
3	Asp 19	0.1	–	–	0
4	Val 20	0.1	–	–	0
5	Tyr 21	2.4	2.4	X	14
6	Gln 22	0.7	0.3	–	2
7	Arg 23	0.2	0.1	–	0
8	Ser 24	0.2	–	–	0
9	Tyr 25	0.5	1.4	X	4

a FoldX and DrugScorePPI predict the penalty in standard binding free energy for substitution of each residue to Ala, the KFC2 server simply predicts hotspots and PCRPi reports the probability that a given residue is a hotspot. Substitutions that are predicted to have a strong effect on the binding are colored in red (i.e., those with a v $\Delta \Delta {\text{G}}_{\text{binding}}^{{}^{\circ}}$ higher than 1.0 kcal/mol or indicated by KFC2 as hot spots or with a PCRPi probability greater than 10). Substitutions that are predicted to have a mild effect on the binding are colored in light red (i.e., those with $\Delta \Delta {\text{G}}_{\text{binding}}^{{}^{\circ}}$ comprised between 0.3 and 1.0 kcal/mol or with a PCRPi probability comprsed between 1 and 10)

#### The Helix of VEGF-C Presents the Most Promising Inter- and Intra-molecular Interactions

**Figure 2:**
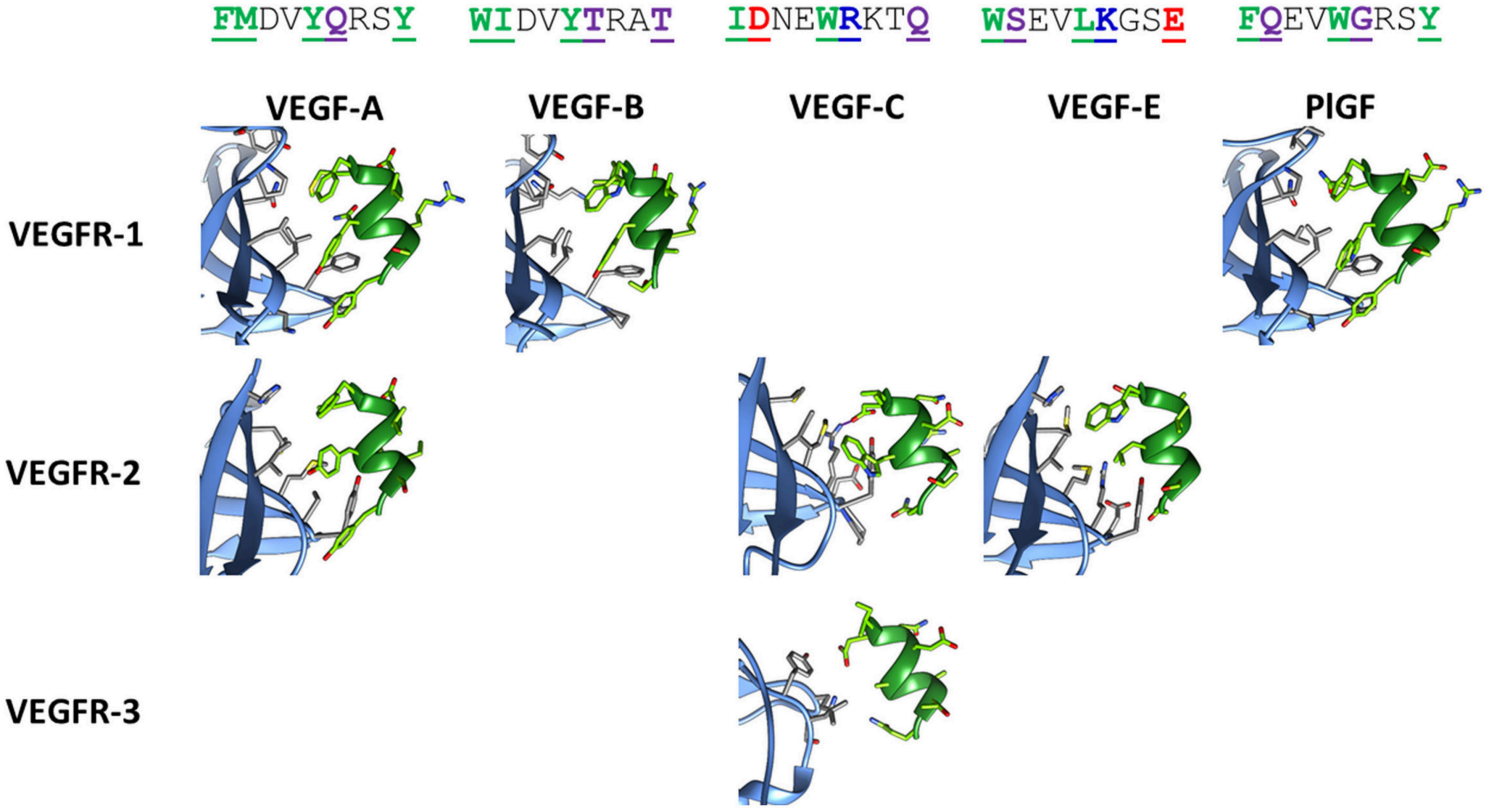
Structures of the helix-interacting interface in VEGF/VEGFR receptor complexes. The receptor is shown in light blue and the growth factor helix in green. The following crystallographic structures were used: 1FLT for VEGF-A/VEGFR-1, 3V2A for VEGF-A/VEGFR-2, 2XAC for VEGF-B/VEGFR-1, 2X1X for VEGF-C/VEGFR-2, 4BSK for VEGF-C/VEGFR-3, 3VSB for VEGF-E/VEGFR-2, 1RV6 for PlGF/VEGFR-1. Sequences of the helix correspond to residues 17–25 for VEGF-A, -B and -E, residues 122–130 for VEGF-C, and residues 25–33 for PlGF. In the sequences, reported on top in the single letter code, interacting residues are underlined, bolded, and colored, according to the following code: green, purple, red, and blue for hydrophobic, polar, anionic, and cationic residues, respectively. The ion bridge formed in the VEGF-C/VEGFR-2 complex is indicated by a purple line.

Figure 2 shows the corresponding helical sequences in various growth factors, together with the available crystallographic structures of their complexes with receptors. In most cases, the interaction is largely based on hydrophobic effects. The only significant exception is provided by VEGF-C, which forms an intermolecular salt bridge with Arg164 of VEGFR-2 through the Asp residue at position 2 of the helix. This specific interaction is predicted to improve the selective recognition of the VEGF-C helix by VEGFR-2. Figure S1 shows the results of an *in silico* Ala scan performed on the VEGFR-2/VEGF-C complex, confirming the centrality of the N-terminal helix in the recognition process.

The VEGF-C helix presents also another interesting property. Short helical segments are usually largely disordered when separated from the protein that contained them. Without the stabilization provided by the rest of the protein structure, competition by water molecules breaks the intramolecular H-bond network that holds the helical conformation together. On the other hand, binding to the receptor requires a helical conformation. Therefore, a disordered peptide pays a significant entropic cost for association to the receptor. Thus, stabilization of the helical conformation in such short peptides is advisable. Interestingly, the helix of VEGF-C presents three pairs of side chains that potentially form interactions stabilizing a helical conformation, being located at an i - i+3 or i - i+4 distance. In particular, Ile1 and Trp5 can form a hydrophobic cluster, while both Asp2 and Arg6 and Glu4 and Lys7 can form salt bridges (numbers correspond to positions in the helix).

**Figure 3:**
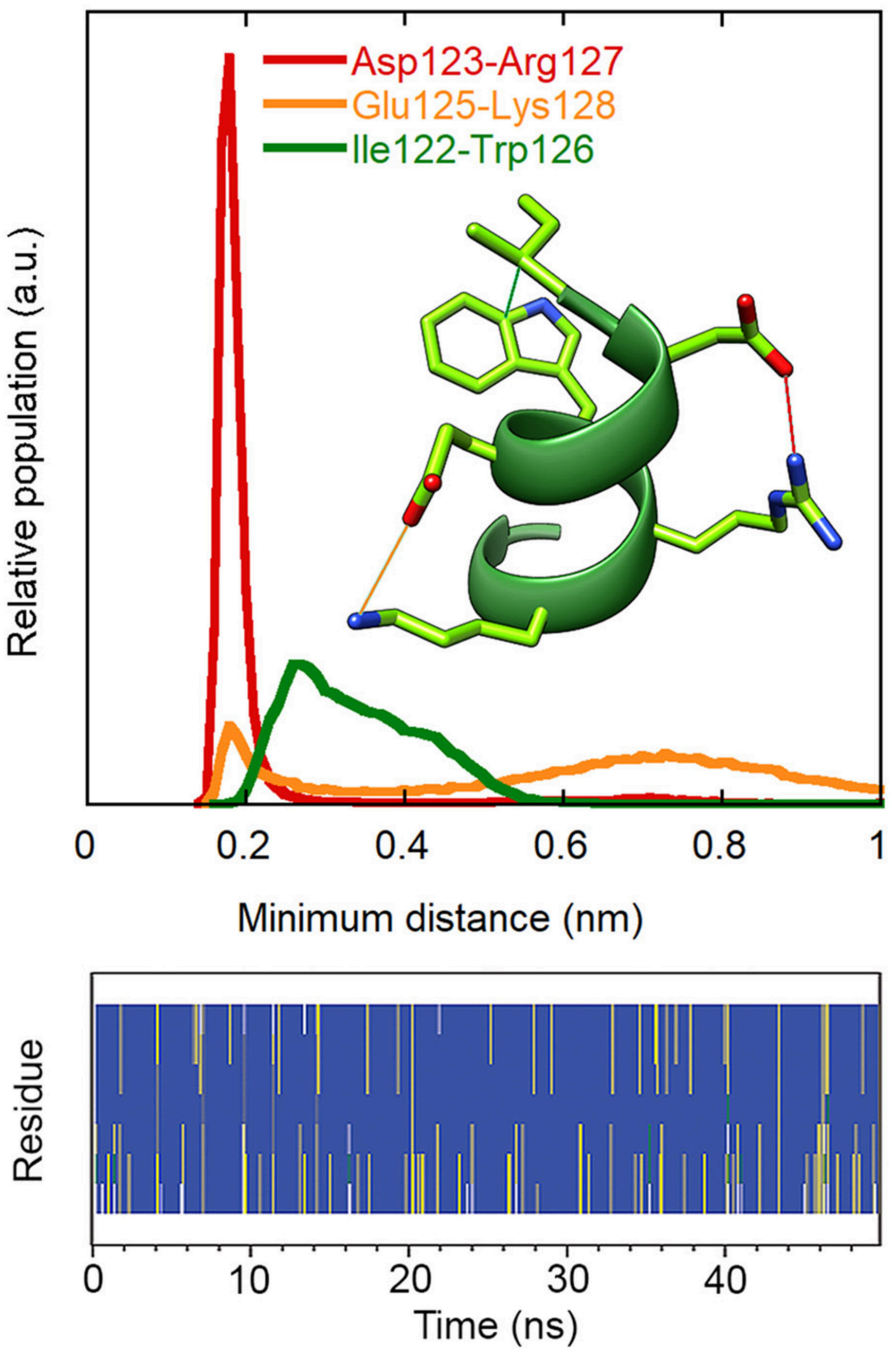
Stability of intramolecular interactions and secondary structure in the REMD simulation of peptide **1** (300 K replica). **Upper:** distributions of the minimum distances between the charged groups of Asp123 and Arg127 (red) and of Glu125 and Lys128 (orange) and between the side chains of Ile122 and Trp126 (green). Residue numbers refer to the VEGF-C sequence. The first ns of the simulation was excluded from the analysis. **Lower:** secondary structure according to DSSP. Blue: alpha helix, grey: 3_10_ helix, yellow: turn, green: bend, white: coil.

Replica exchange molecular dynamics (REMD) simulations were used to verify the stability of the VEGF-C helix, when free in solution. Figure 3 reports the secondary structure of the peptide, a representative conformation and the histograms of the distances between the side-chains mentioned above during the simulation trajectory. The data confirm that the ion pairs and hydrophobic cluster are significantly populated and stabilize the helical conformation of the peptide in solution.

**Figure 4:**
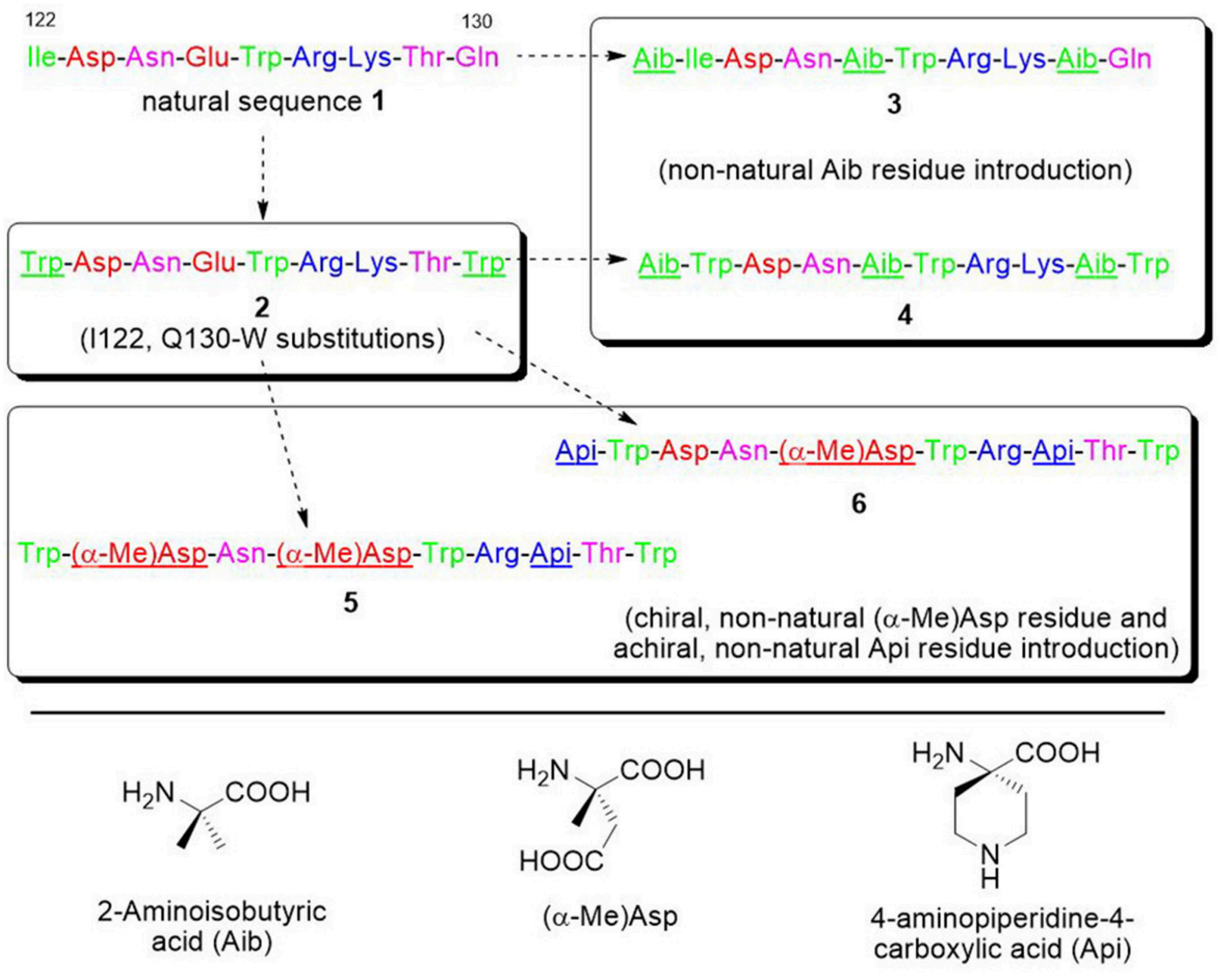
Sequences of the VEGF-C natural portion **1** and of the derived peptides **2**–**6**. Sequences are colored in green, purple, red, and blue for hydrophobic, polar, anionic, and cationic residues. The introduced modifications are underlined.

For all these reasons, we decided to focus on the VEGF-C helix sequence (peptide **1**) (Figure 4) to develop VEGF/VEGFR interaction inhibitors.

#### Substitution of Two Residues in the VEGF-C Sequence Is Predicted to Increase Binding Affinity

**Figure 5:**
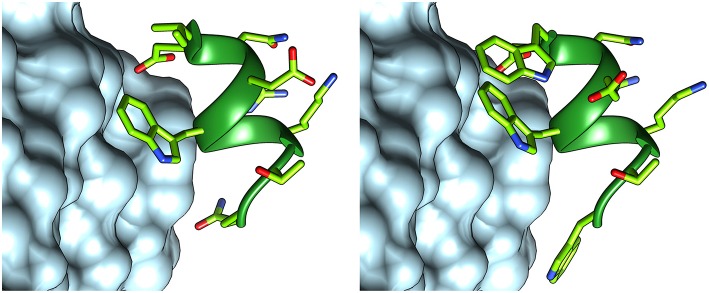
Structures of peptides **1 (left)** and **2 (right)**, interacting with VEGFR-2. The structure of peptide **1** was taken from the VEGF-C structure (PDB code 2X1X), while that of peptide **2** was modeled with FoldX.

In order to optimize peptide affinity for VEGFRs, *in silico* mutagenesis was performed, concentrating on the VEGF-C/VEGFR-2 complex (PDB code 2X1X). All residues of the helix previously identified as hot spots were mutated to all possible coded amino acids, evaluating the effect of the substitution on the standard binding free energy. This analysis identified substitution of the first residue from Ile to Trp, and of the last one from Gln to Trp or Val as the only mutations that would lead to a significant predicted improvement in standard binding free energy (>1 kcal/mol). Either hydrophobic substitution at position 1 would maintain the helix-stabilizing cluster identified above, but Trp has a higher intrinsic helix propensity than Ile (Chakrabartty et al., 1994; Pace and Scholtz, 1998). Based on these findings, sequence **2** was designed (Figure 4). Figure 5 shows how the N- and C-terminal Trp residues increase the interactions between helix and receptor.

A more comprehensive computational analysis on the proposed substitutions, carried out by molecular mechanics—Poisson Boltzmann surface area calculations (MM-PBSA) (Hou et al., 2010), confirmed the prediction, showing that the presence of Trp residues at each peptide terminus should indeed lead to an increased binding affinity (Table 2).

**Table 2:** Results of the MM-PBSA calculations of the interaction of peptides **1** and **2** with VEGFR-2^a^.

**Peptide**	**ΔE_pot_ (kcal/mol)**	**ΔG°_PB_(kcal/mol)**	**ΔSAS (nm^2^)**	**ΔG°*_*binding*_* (kcal/mol)**
**1**	−32 ± 1	26 ± 1	−3.59 ± 0.02	−8 ± 2
**2**	−39 ± 1	27 ± 1	−4.42 ± 0.02	−14 ± 2

a *The various terms are described in the Methods section*.

#### C^α,α^-Disubstituted Amino Acids Can Be Inserted to Increase Peptide Helicity and Stability

C^α,α^-disubstituted α amino acids strongly stabilize helical structures: due to steric interactions between the gem alkyl and methyl groups linked to the α-carbon, the accessible conformational space of such residues is extremely limited and is located in the region of the Ramachandran plot corresponding to helical structures. For this reason, such C^α,α^-disubstituted amino acids constitute suitable building blocks to synthesize oligopeptide with a stable helical folding (Toniolo et al., 2001). In addition, insertion of non-proteinogenic amino acids strongly reduces peptide susceptibility to proteolysis (De Zotti et al., 2009). The simplest and most studied C^α,α^-disubstituted amino acid residue is α-amino isobutyric acid or Aib. Aib is a natural amino acid featuring two methyl groups on its α-carbon. It is non-ribosomally included in peptide sequences by fungal synthases. This achiral residue is a well-known helix inducer. One peptide sequence modified to include Aib is the well-known commercial drug semaglutide (Al Musaimi et al., 2018). Positions not involved in the interaction (i.e., 3, 4, 7, and 8 in the helix, see above) were considered for possible modifications. Position 2 was analyzed as well, as the C^α,α^-disubstituted amino acids analogue of Asp, α-methyl-aspartic acid (α-Me)Asp, is commercially available. Although literature reports on the conformational properties of this specific residue are lacking, the preference for helical conformations is a common feature of methyl-containing disubstituted residues, and its homologue (α-Me)Asn was reported to promote type III β turn (i.e., a portion of a 3_10_ helix) in short peptides (Hopkins et al., 2000). Analysis of the structure of the VEGF-C/VEGFR-2 complex showed that addition of a methyl group on the alpha carbon at these positions, or addition of a residue at the N-terminus, would not cause any intermolecular clashes.

Based on these considerations, four analogues were designed, based on sequence **2** (Figure 4). In analogues **5** and **6**, Glu4 was substituted by (α-Me)Asp, and Lys7 by the cationic amino acid Api (4-aminopiperidine-4-carboxylic acid). Such cyclic residue can promote a helical conformation to some extent when incorporated into peptides (Cho et al., 2010; Dalzini et al., 2016). In addition, in peptide **5** Asp2 was substituted by (α-Me)Asp, too, while in **6** an Api residue was added at the N-terminus. In analogue **4**, Aib was inserted at positions 4 and 8, and also added at the N-terminus. For comparison, analogue **3** has been synthesized, with the same sequence of **4**, but without the Trp substitutions.

### Synthesis of VEGF-C Derived Peptides 1-6

Peptide sequences **1**–**6** (Figure 4) were conveniently prepared by solid-phase peptide synthesis (SPPS) on Rink Amide 4-methylbenzhydrylamine (MHBA) resin using the 9-fluorenylmethoxycarbonyl/*t*-butyl ether (Fmoc/*t*Bu) strategy. Each step of the SPPS was performed with a semi-automatic synthesizer, assisting coupling reactions with microwaves. First, the resin was swelled in dimethylformamide (DMF) and treated with a 25% solution of piperidine in DMF to remove the Fmoc-protecting group, releasing the reactive amino moiety on the beads (step a). The Fmoc-amino acid to be attached to the solid support was activated with *N,N*′-diisopropylcarbodiimide (DIC) and 1-hydroxy-7-azabenzotriazole (HOAt) coupling reagents in the presence of *N,N*-diisopropylethylamine (*i*Pr_2_NEt) in DMF: after stirring for 25 min at 0°C, the mixture was added to the resin and a cycle of coupling, capping and deprotection was performed (step b). This procedure was repeated until the sequence was completed, then the N-terminal residue was acetylated and the beads were treated with TFA in the presence of thioanisole, 1,2-ethanedithiol (EDT) and anisole as scavengers. Subsequent purification of the crude peptide with reversed phase high-performance liquid chromatography (RP-HPLC) and freeze-drying from glacial acetic acid gave the pure compound as a fluffy solid.

Due to the high steric congestion, α,α-disubstituted residues are poorly reactive even after activation. For this reason, the coupling reaction was performed twice whenever a quaternary amino acid had to be attached to the resin: according to this synthetic protocol, after the first coupling step, a second aliquot of the activated quaternary amino acid was added to the resin and another condensation reaction was carried out. This procedure allowed to minimize the number of unreacted amino moieties on the beads.

Further details are provided in the Supplementary Information (Figures S2–S14, Tables S1–S6, Scheme S1).

### Structural Investigation

**Figure 6:**
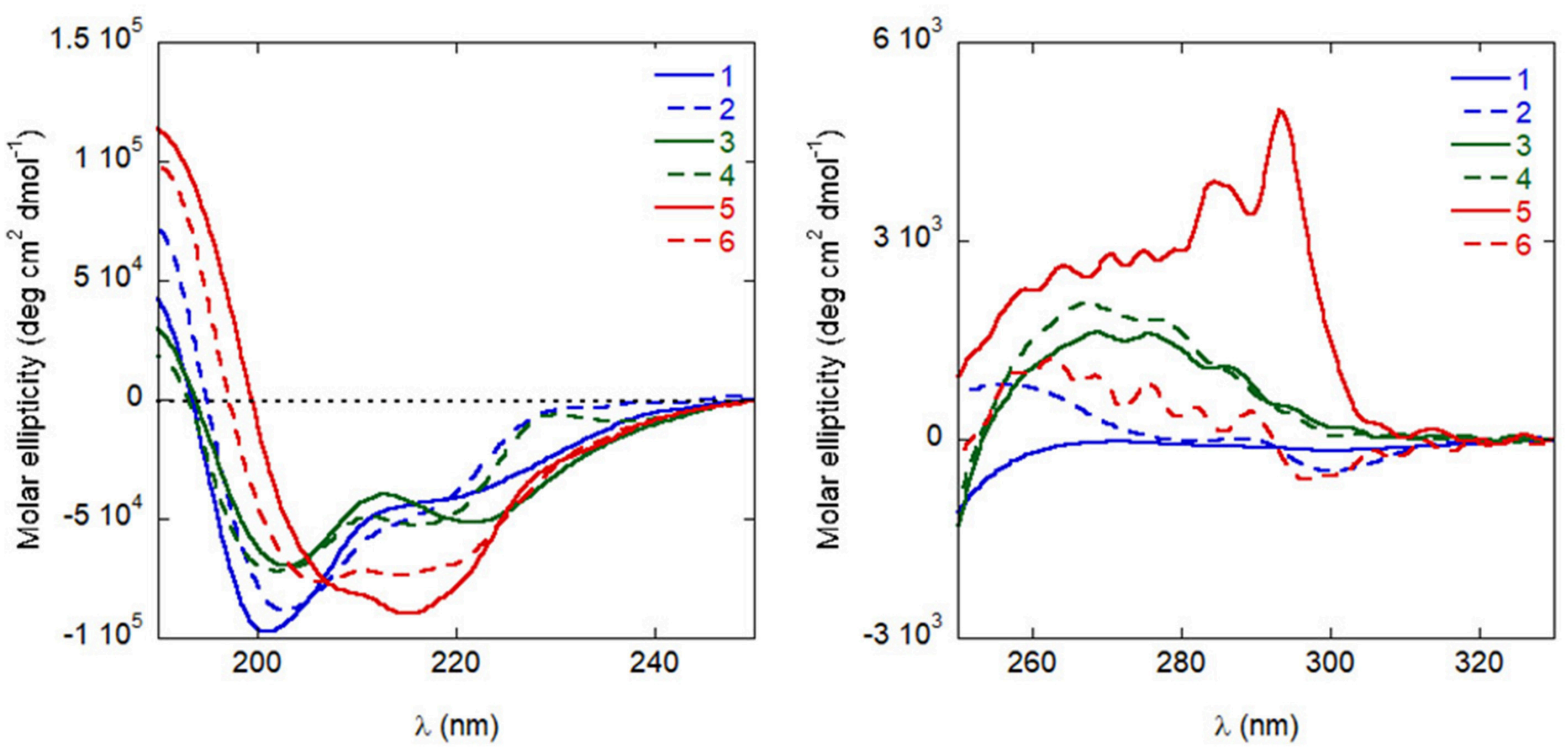
CD spectra of the peptides in distilled H_2_O (10^−4^ m), in the far UV **(left)** and near UV **(right)**.

Circular dichroism (CD) experiments in water demonstrated that all analogues populate helical conformations in solution to some extent, as predicted by REMD simulations for the natural sequence **1**. As shown in Figure 6, the positive peak at approximately 190 nm, and the negative bands at about 205 and 220 nm, typical of helical structures, are present in the spectra of all analogues (Kelly and Price, 1997). Quantitative determinations of the degree of helicity is complicated by the presence of multiple aromatic residues, whose side-chains can contribute significantly to the CD in the far UV region. However, several features indicate that analogues **5** and **6** are more helical than the other peptides: compared to the spectra of analogues **1**–**4**, the positive peak is higher, the negative band at short wavelengths is red-shifted and the ratio of the two negative bands at approximately 220 and 205 nm is closer to one (Kelly and Price, 1997). Actually, for **5** this ratio is even higher than one, possibly indicating that some peptide aggregation is taking place (Dai et al., 2004). A stabilizing effect of the introduced C^α,α^-disubstituted amino acids was less obvious in the spectra of analogues **3** and **4**.

**Figure 7:**
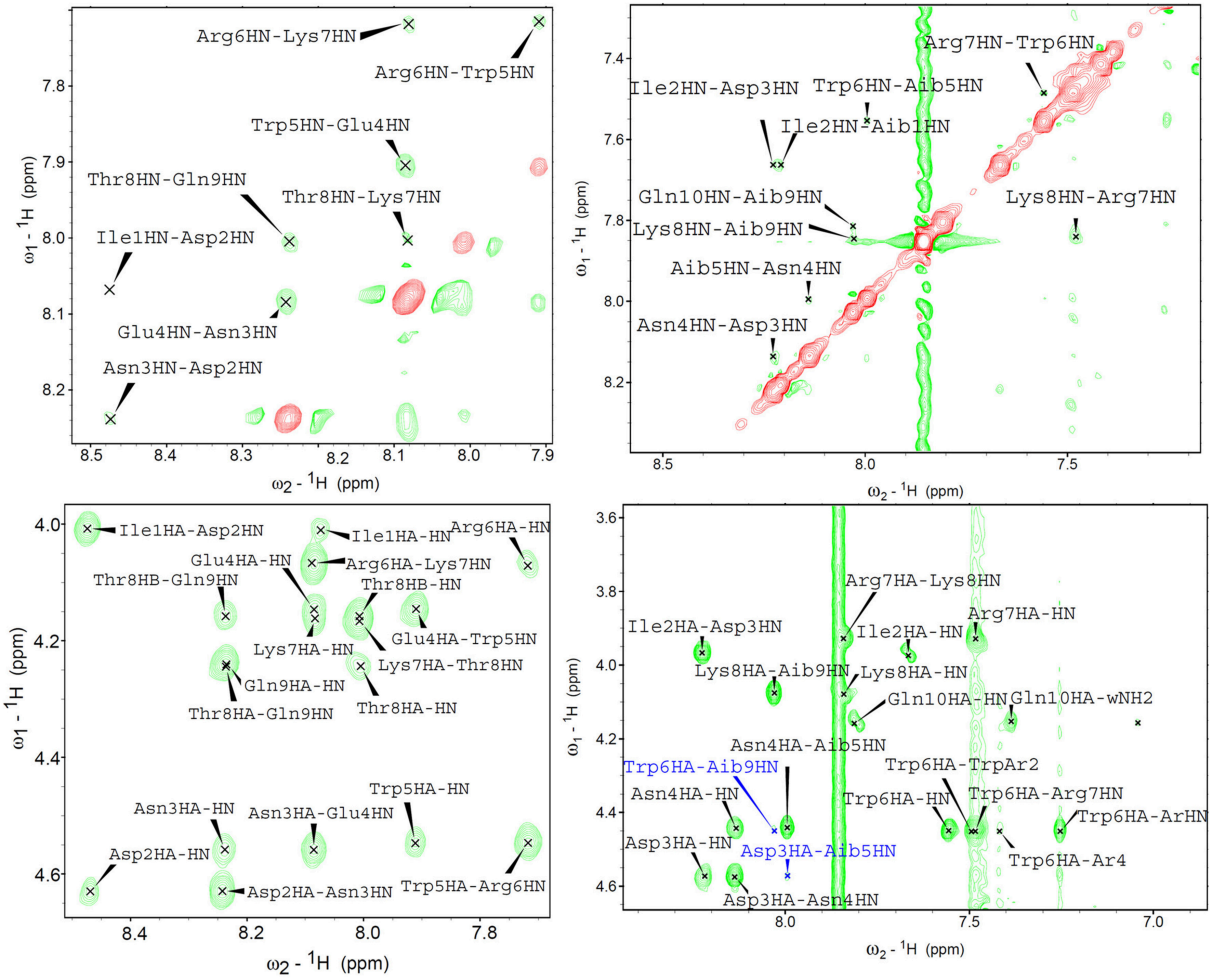
2D proton NMR ROESY spectra in H_2_O:D_2_O 9:1 (600 MHz, 298K) for compounds **1 (left)** and **3 (right)** (concentrations 1.1 and 1.4 mm, respectively). Top panels report NH-NH sequential correlations, while the bottom panels correspond to the fingerprint region showing long-range αH-NH correlations. In the case of compound **3**, long range cross-peaks, including the αH_*i*_ → NH_*i*+2_ diagnostic of 3_10_-helical structures, are highlighted in blue.

In near UV CD spectra, the induced dichroism band of Trp was more intense for analogues **3**–**6** than for peptides **1** and **2**, which comprise coded amino acids only. This finding indicates that the C^α,α^-disubstituted amino acids rigidified the helical conformation. To confirm this conclusion also in the case where far UV CD spectra were less informative, analogues **1** and **3** were directly compared in 2D nuclear magnetic resonance (NMR) measurements in water (Figure 7). For both peptides, all sequential NH-NH cross peaks were present in the ROESY (Rotating-frame nuclear Overhauser Effect correlation SpectroscopY) spectrum, indicating the onset of a helical structure. However, the spectra did not show any long range connectivity for **1**, while the presence of two long-range cross-peaks (Asp3HA-Aib5HN and Trp6HA-Aib9HN) for peptide **3**, including an αH_i_ → NH_i+2_ interaction, is a clear indication that this analogue adopts a well-developed 3_10_-helical conformation in water.

Overall, these data indicate that the introduction of C^α,α^-disubstituted amino acids stabilized the helical conformations, particularly in analogs **5** and **6**, where chiral (α-Me)Asp residues were inserted in the sequence.

### Biological Studies

#### Inhibition of VEGF-A/VEGFR-1 Complex Formation *in vitro*

A chemiluminescent assay to determine the inhibition of complex formation between VEGF-A (isoform VEGF_165_) and the extracellular domain of VEGFR-1 (Goncalves et al., 2007; García-Aranda et al., 2013) had been previously employed in our group (Zanella et al., 2015). VEGF-C (from which analogue **1** was derived) is not selective for VEGFR-1 (Su et al., 2007), but unfortunately the same assay protocol turned out to be ineffective with VEGFR-2, at least in our hands. The assay based on VEGFR-1 was therefore applied here as a very stringent test of the affinity of our peptides for VEGFRs, and to assess the different peptides comparatively. All peptides were able to inhibit complex formation (Table 3), although in the high micromolar range. The introduction of quaternary amino acids resulted in a beneficial effect in terms of affinity toward VEGFR-1, with analogs **3**, **5** and **6** being the most effective. Further studies were focused on analogues **5** and **6**, considering also the higher stability of their secondary structure, which affects resistance to proteolytic degradation.

**Table 3:** Inhibition of biotinylated VEGF_165_ binding to isolated VEGFR-1.

**Peptide**	**% Inhibition^a^**
1	27 ± 1
2	29 ± 7
3	86 ± 1
4	40 ± 10
5	82 ± 1
6	83 ± 3

a *% of inhibition of biotinylated VEGF_165_ binding to VEGFR-1 at 500 μM*.

#### Resistance to Proteolytic Degradation

Peptides **5** and **6** were very stable against proteases, presumably thanks to the presence of non-natural amino acids in their sequences and to their stable secondary structure. HPLC analysis (Figures S15–S24) demonstrated that peptide **5** was fully stable to trypsin, chymotrypsin and pronase for 90 minutes and it persisted even after many days, although some degradation was slowly occurring. Peptide **6** was fully stable even after 6 days. By contrast, the natural sequence **1** was degraded within 15 min by all three enzymes.

#### Inhibition of HUVEC Morphogenesis

**Figure 8:**
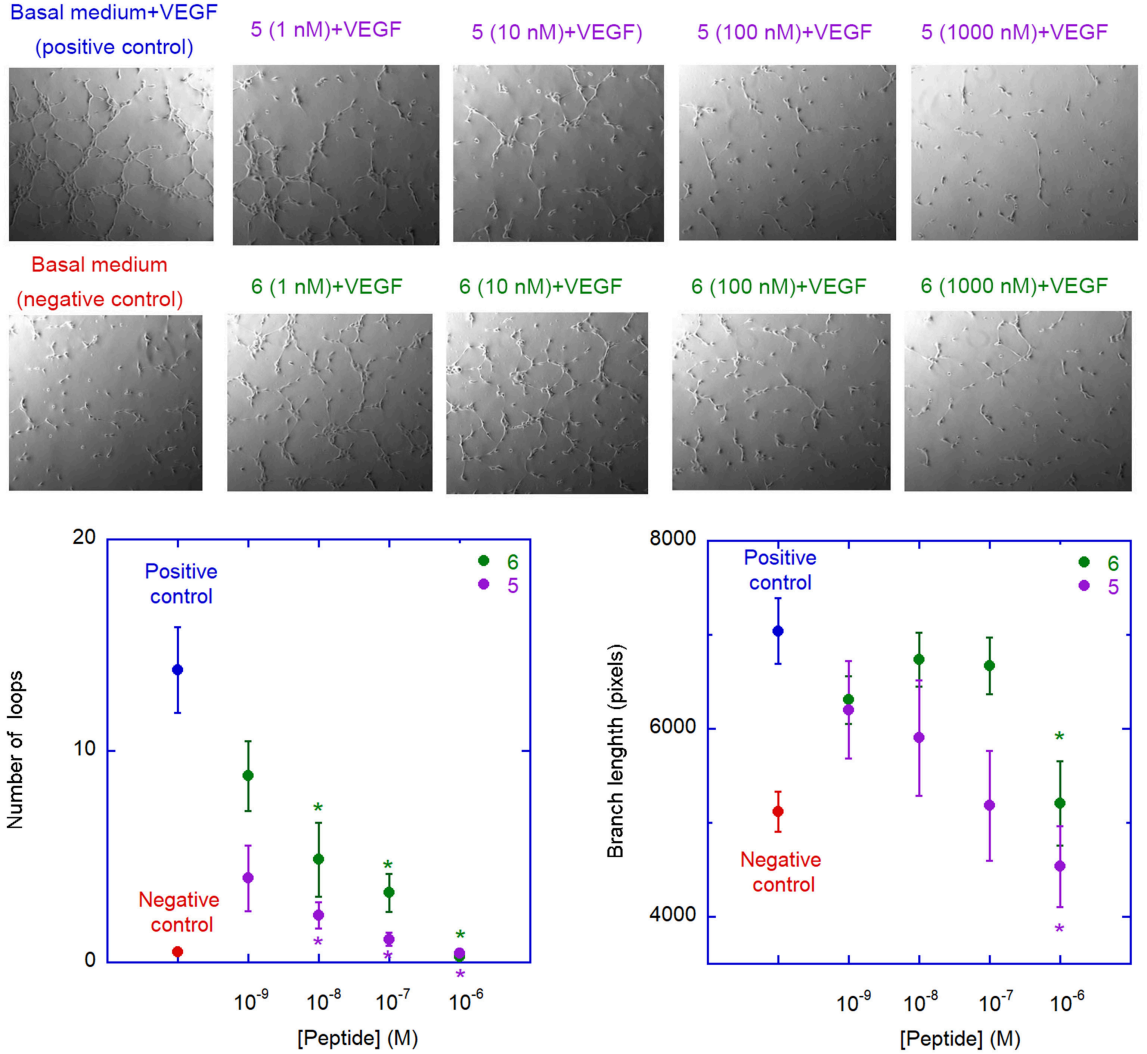
Antiangiogenic activity of analogues **5** and **6**. The images are representative phase contrast photomicrographs of HUVECs plated on Matrigel under resting conditions (lower left) or after stimulation with VEGF_165_, 10 ng/mL (upper left); the effect of VEGF on loop formation, in comparison to medium alone, is clearly evident in the upper panel. **5** (upper panels) and **6** (lower panels) were added at different concentration on VEGF-stimulated cells; both **5** and **6** are able to reduce, in a concentration-dependent manner, the ability of VEGF to induce loops formation. The graphs describe the effect of addition of **5** (purple) and **6** (green) on VEGF-induced neovessel formation measured as total number of loops (left graph) or total branch length (right graph). Data are expressed as mean ± SEM of 5 separate experiments. Asterisks indicate significant differences with respect to the positive control (basal medium + VEGF), with *p* < 0.05, according to a two-tailed paired *t*-test. Negative controls (basal medium only) are also shown.

The ability of peptides **5** and **6** to affect neovessel formation *in vitro* was investigated on HUVECs according to the previously reported procedure (Fanelli et al., 2014). Experiments were performed in the presence of peptide **5** or **6** under resting (absence of stimuli) or stimulated (VEGF_165_) conditions. As expected, under resting conditions, HUVECs did not show any significant network formation, while the presence of VEGF_165_ induced a strong increase in loop and branches formation (Figure 8). Pre-incubation with either **5** or **6** significantly reduced VEGF_165_-induced loop and branches formation and this effect was concentration-dependent, reaching statistical significance at 10 nm for loop and 1 μm for branches formation (Figure 8). Interestingly, these values are much lower than the concentrations needed to inhibit VEGF-A/VEGFR-1 association, possibly indicating a significant selectivity toward VEGFR-2. It is worth noting that inhibition of loop formation was more marked for analogue **5** than for **6**, at all concentrations tested.

## Discussion

Several peptides able to bind to VEGFRs have been reported in the literature. Some of them were identified by library screening or phage display approaches, while others were developed by rational design, reproducing different epitopes of the VEGFR interacting surface of VEGFs. However, most of these studies were focused on mimicking VEGF-A, which is traditionally considered the master regulator of angiogenesis through VEGFR-2 binding (Ferrara and Adamis, 2016). In particular, all helical foldamers mimicking the N-terminal helix were developed based exclusively on the interacting elements of VEGF-A, i.e., hydrophobic residues only. Examples include the MA peptide (Diana et al., 2013) and peptides developed by Pérez De Vega and coworkers (García-Aranda et al., 2013; Balsera et al., 2017). Due to their hydrophobic driving force for association, these peptides are prone to selectivity issues, since binding sites for amphipathic helical peptides are extremely common at protein-protein interfaces (Bonache et al., 2014). It is also worth mentioning that some of the peptides modeled on the VEGF-A helix exhibited pro-angiogenic, rather than anti-angiogenic, activity (De Rosa et al., 2018).

In the present study, from an analysis of the available crystallographic structures of VEGFs/VEGFRs complexes, we noted that the same helix in VEGF-C exhibits several interesting features, including an intermolecular salt bridge with VEGFR-2, and multiple intramolecular helix-stabilizing interactions. For these reasons, we focused our study on the VEGF-C sequence.

VEGF-C binds to both VEGFR-2 and VEGFR-3 (Su et al., 2007; Leppänen et al., 2010; Chen et al., 2013; Wang and Tsai, 2015). Traditionally, regulation of lymphangiogenesis through association with VEGFR-3 has been considered its prevalent activity. Indeed, VEGF-C expression is closely related to lymphangiogenesis and lymphatic metastasis in a variety of human tumors (Chen et al., 2013). However, more recently, VEGF-C has been demonstrated to play an important role also in the regulation of physiological and pathological angiogenesis (Chen et al., 2013). In addition, it has fundamental functions in the autocrine signaling of cancer cells. VEGF-C is expressed in a number of human malignancies, and high levels of expression correlate with poor prognosis (Chen et al., 2013). Autocrine VEGF-C signaling regulates cell invasion, proliferation, and resistance to chemotherapy (Su et al., 2007). VEGF-C can also modulate the immune system so that tumor cells more easily escape immune surveillance (Wang and Tsai, 2015). A very recent, groundbreaking article (Michaelsen et al., 2018; Niclou, 2018), identified VEGF-C, rather than VEGF-A, as the main responsible for autocrine VEGFR-2 activation and cell proliferation in glioblastoma. In this study, VEGF-C silencing was superior to bevacizumab therapy in improving tumor control. Interestingly, bevacizumab treatment increased VEGF-C expression (Michaelsen et al., 2018), and up-regulation of VEGF-C has been observed in tumor cells that have acquired resistance to anti-VEGF-A therapy (Wang and Tsai, 2015), suggesting that VEGF-C may compensate for VEGF-A depletion. For all these reasons, molecules inhibiting VEGF-C signaling, rather than, or in addition to VEGF-A interactions, might find important therapeutic applications. In principle, our peptides, based on the VEGF-C helix, can compete with both growth factors for binding to the receptors.

The VEGF-C nonapeptide helix sequence has some intrinsic helix propensity, but CD and NMR studies demonstrated that insertion of C^α,α^-disubstituted amino acids led to a significant stabilization of the secondary structure. In particular, analogues **5** and **6**, containing the chiral (α-Me)Asp amino acids, had a more stable helical conformation than peptides **3** and **4**, where achiral Aib residues were introduced. Peptides comprising these modifications were highly resistant to proteolytic degradation, and stabilization of the helical conformation probably contributed to favor receptor binding, by reducing the entropic cost of the association process. Indeed, these molecules were able to bind even receptor VEGFR-1, where specific ion-pair interactions are not possible, although in the high micromolar range. More importantly, they inhibited VEGF-induced morphogenesis in the low nm range, possibly through their interaction with the VEGFR-2 receptor.

One point worth mentioning is that the N-terminal VEGF helix, mimicked by our peptides, is involved also in the interface of the growth factor dimer (see Figure 1). Therefore, inhibition of VEGF dimerization could contribute to the antiangiogenic activity of the compounds developed here.

Further studies are warranted to characterize the interaction of the designed peptides with different VEGFRs and the molecular mechanisms of their antiangiogenic activity. However, our data clearly indicate that the development of antiangiogenic folded synthetic peptides inspired by the VEGF-C N-terminal helix might open the way to a novel class of anticancer agents.

## Methods

### *In silico* Mutagenesis

*in silico* alanine scanning was performed with FoldX 4.0 (Schymkowitz et al., 2005) and the DrugScorePPI (Krüger and Gohlke, 2010), KFC2 (Zhu and Mitchell, 2011), and PCRPi (Segura Mora et al., 2010) servers. *In silico* mutagenesis was performed with FoldX, by first repairing the PDB file (PDB code 2X1X), then introducing the mutations and finally calculating the complex standard binding free energy, and subtracting the value of the WT complex. Structural images were obtained using the Chimera software (Pettersen et al., 2004).

### Molecular Dynamics Simulations

Molecular dynamics (MD) simulations were carried out by using the GROMACS 4.6.3 software package (Hess et al., 2008). The ff53a6 parameters (Oostenbrink et al., 2004) were adopted for the complexes and the SPC model (Berendsen et al., 1981) was used for the water molecules. Short-range electrostatic interactions were cut-off at 1.2 nm and long range electrostatic interactions were calculated using the particle mesh Ewald (PME) algorithm (Essmann et al., 1995). Simulations were run with a 2 fs time step. The Berendsen algorithm was used to keep temperature and pressure constant (Berendsen et al., 1984). Bond lengths were constrained with the LINCS algorithm (Hess et al., 1997). The structure with PDB code 2X1X from the Protein Data Bank was used as a starting point for the simulations. After solvating the proteins with roughly 6500 water molecules and an opportune number of Na^+^ and Cl^−^ ion to ensure electroneutrality in a box of about 180 nm^3^, the potential energy of the systems was minimized. A multi-step procedure was adopted for equilibration, first constraining both the protein and the peptide, then releasing the constraints on the peptide conformation, and finally removing all constraints. In each step, the temperature was gradually raised from 50 K to 300 K in 8 ns, and the conformation with the most favorable peptide-protein interaction energy was selected as the starting structure for the successive step. Finally, 50 ns production runs were performed.

### MM-PBSA Calculations

The protein-peptide binding energies were obtained from MD simulations by using the MM-PBSA protocol. The binding free energies were calculated according to the following equation

$$\begin{array}{l} \Delta G_{binding}^{{}^{\circ}}=G_{complex}^{{}^{\circ}}-G_{receptor}^{{}^{\circ}}-G_{ligand}^{{}^{\circ}} \end{array}$$

In these free energy values, the potential energy terms were obtained by using the GROMACS g_energy tool. The electrostatic solvation terms were calculated with the APBS software (Baker et al., 2001). The dielectric constant was set equal to 78.54 for water and 2 for the solute. The input files for APBS were generated with the PDB2PQR server (Dolinsky et al., 2004).

The non-polar solvation energies were computed as

$$\begin{array}{l} G_{\text{surf}}^{{}^{\circ}}=\gamma SAS \end{array}$$

in which γ was set equal to 0.543 kcal mol^−1^ nm^−2^ (Fogolari et al., 2003), solvent accessible surface (SAS) values were obtained by using the g_sas tool of GROMACS.

### REMD Simulations

REMD simulations were performed using the GROMACS software package. An extended conformation was used as the starting structure. After minimization and equilibration, 16 replicas were simulated with temperatures ranging from 260 to 650 K, chosen to ensure an exchange probability between replicas equal to 55% (Patriksson and van der Spoel, 2008); exchanges were attempted every 2 ps and the simulation time of each replica was 50 ns. The Amber FF99-SB parameters were used to describe the peptide (Hornak et al., 2006) and the OBC (Onufriev, Bashford, and Case) GBSA implicit solvent model (Onufriev et al., 2004) was used to mime the solvent. Bond lengths were constrained with the SHAKE algorithm (Ryckaert et al., 1977) and simulations were run with a 2-fs time step. Temperatures were kept constant by using the velocity-rescale algorithm (Bussi et al., 2007). Secondary structure was assigned by means of Dictionary of Protein Secondary Structures (DSSP) (Kabsch and Sander, 1983). The g_mindist tool in GROMACS was used to calculate the minimum distances between groups of atoms.

### SPPS

Semi-automatic SPPS was accomplished through the Biotage® Initiator™ synthesizer, assisted by microwave (MW) irradiation; Fmoc/*t*Bu strategy and Rink Amide MHBA Resin (100–200 mesh; loading: 0.5 mmol/g) were used. Peptides were obtained with yields up to 30% and purities between 97% and >99%. Refer to the Supporting Information for a detailed description of materials, procedures, and methods.

### CD

Jasco J-715 (Tokyo, Japan) spectropolarimeter—equipped with a Haake thermostat (Thermo Fisher Scientific, Waltham, MA)—was used to collect circular dichroism spectra. Bidistilled water was used as solvent. Fused quartz cells of either 1 or 10 mm pathlength (Hellma, Mühlheim, Germany) were used. Spectra were baseline subtracted as expressed in terms of [θ]_T_, total molar ellipticity (deg × cm^2^ × dmol^−1^).

### NMR

2D-NMR experiments for conformational studies were carried out on a Bruker Avance DMX-600 instrument, using TOPSPIN 1.3 software package. For peptide **3**, COSY (COrrelation SpectroscopY), CLEAN-TOCSY (TOtal Correlation SpectroscopY), and ROESY spectra (all with watergate for water suppression) were acquired. COSY was phase sensitive. TOCSY spectrum (spin-lock pulse, 70 ms) was acquired by collecting 400 recordings of 76 scans each. For the ROESY spectrum (200 ms mixing time), 512 experiments, each one consisting of 80 scans, were acquired. The full assignment was achieved exploiting the procedure proposed by K. Wüthrich (Wüthrich, 1986).

### Inhibition of VEGF/VEGFR Complex Formation *in vitro*

The chemiluminescent screening assay for the detection of VEGFR-1 ligands was accomplished according to the procedure described in the Supporting Information. Unlabeled VEGF_165_, employed as reference compound, showed an IC_50_ value of 146 pm (Zanella et al., 2015), comparable with the previously reported value (Goncalves et al., 2007).

### Inhibition of HUVEC Morphogenesis

To assess angiogenic activity, HUVECs (2.5 × 10^4^ cells) were seeded in a 24-well plate coated with 100 μL/well of Matrigel previously polymerized for 1 h at 37°C. Cells were then incubated for 5 h at 37°C in a moist atmosphere of 5% CO_2_. The experiments were performed without or in the presence of **5** and **6** under either resting (absence of stimuli, cell cultured in EndoGRO medium alone, without FBS and all the growth factors) or stimulated conditions (addition of VEGF_165_, 10 ng/mL). Network formation was evaluated by phase-contrast microscopy using a fluorescence microscope (AxioVert 40CFL, Carl Zeiss S.p.A. Milan, Italy). Five photos of each well were recorded with 10X magnification. Network formation was finally quantified in terms of mean number of loops per field as topological parameters and the total length of the branches. For the purpose of the analysis, loops were defined as any complete ring formed by HUVECs, while open ramifications were considered as branches. The analysis of the images was performed using the free software ImageJ (https://imagej.nih.gov/ij/).

### Resistance to Proteolytic Degradation

The proteolytic stability of peptides **1**, **5**, and **6** was assessed against three enzymes: pronase, trypsin and chymotrypsin (Sigma-Aldrich). Each peptide was dissolved in the appropriate buffer [i.e., (2-amino-2-hydroxymethyl-propane-1,3-diol (Tris)·HCl 20 mm, containing 20 mm CaCl_2_ pH 7.6 for pronase; Tris·HCl 50 mm, pH 7.8 for trypsin and chymotrypsin). Then, it was incubated with and without the enzyme solution (1.25 μg of enzyme in 150 mL buffer) at 37°C for 12 h. The mixture was analyzed by RP-HPLC (column: Phenomenex Jupiter 5 μ C18 300 Å) every ten minutes for the first hour, then every half hour. Gradients: 5–25%B in 10 min for peptide **1**; 15–35%B in 10 min for peptide **5**; 15–25%B in 10 min for peptide **6**. Eluants: A, H_2_O:CH_3_CN 9/1 + 0.05% trifluoroacetic acid (TFA); B: CH_3_CN:H_2_O 9/1 + 0.05% TFA.

## Data Availability

All datasets generated for this study are included in the manuscript and/or the Supplementary Files.

## Author Contributions

SZ, LP, and GS synthesized the peptides. GB, together with SR, AP, and LS, performed the *in silico* studies. MD carried out the conformational studies. DA designed and performed the binding experiments and FM determined the biological activity of the synthesized compounds. AS performed proteolytic stability assays. LB and UP supervised the binding and activity studies, FF the structural characterization, CG the peptide synthesis, LS conceived the original idea and supervised peptide design. SZ, LB, and LS wrote the manuscript. All authors discussed the results and commented on the manuscript.

### Conflict of Interest Statement

The authors declare that the research was conducted in the absence of any commercial or financial relationships that could be construed as a potential conflict of interest. The reviewer NI declared a past co-authorship and collaboration with one of the authors (LS).
