# Supplementary material for: Rational Design of Antiangiogenic Helical Oligopeptides Targeting the Vascular Endothelial Growth Factor Receptors
Source: Front Chem. 2019 Mar 29;7:170. doi: 10.3389/fchem.2019.00170 (PMC6449863; doi:10.3389/fchem.2019.00170)
Supplement: Supplementary file 1 [file Data_Sheet_1.PDF]

SUPPLEMENTARY INFORMATION

Rational Design of Antiangiogenic Helical Oligopeptides Targeting the Vascular Endothelial Growth Factor Receptors

Simone Zanella[1§], Gianfranco Bocchinfuso[2§], Marta De Zotti[3§], Daniela Arosio[4], Franca Marino[5], Stefano Raniolo[2], Luca Pignataro[1], Giovanni Sacco[1], Antonio Palleschi[2], Alvaro S. Siano[6], Umberto Piarulli[5], Laura Belvisi[1,4], Fernando Formaggio[3*], Cesare Gennari[1,4*], Lorenzo Stella[2*]

[1]University of Milan, Department of Chemistry, 20133 Milan, Italy
[2]University of Rome Tor Vergata, Department of Chemical Sciences and Technologies, 00133 Rome, Italy
[3]University of Padova, and ICB CNR, Padova Unit, Department of Chemistry, 35131 Padova, Italy
[4]National Research Council (CNR), Institute of Molecular Science and Technologies (ISTM), 20133 Milan, Italy
[5]University of Insubria, Center for Research in Medical Pharmacology, 21100 Varese, Italy
[6]Universidad Nacional del Litoral (UNL), Departamento de Química Organica, Facultad de Bioquímica y Ciencias Biologicas (FBCB), 3000 Santa Fe, Argentina

Table of Contents

IN SILICO ALA SCAN 3
MATERIALS AND METHODS 4
GENERAL PROCEDURES FOR SEMI-AUTOMATIC SPPS 5
SYNTHESIS OF VEGF-C DERIVED PEPTIDES 1-6 8
HPLC CHROMATOGRAMS 15
MS SPECTRA 18
ENZYMATIC DEGRADATION 21

IN SILICO ALA SCAN

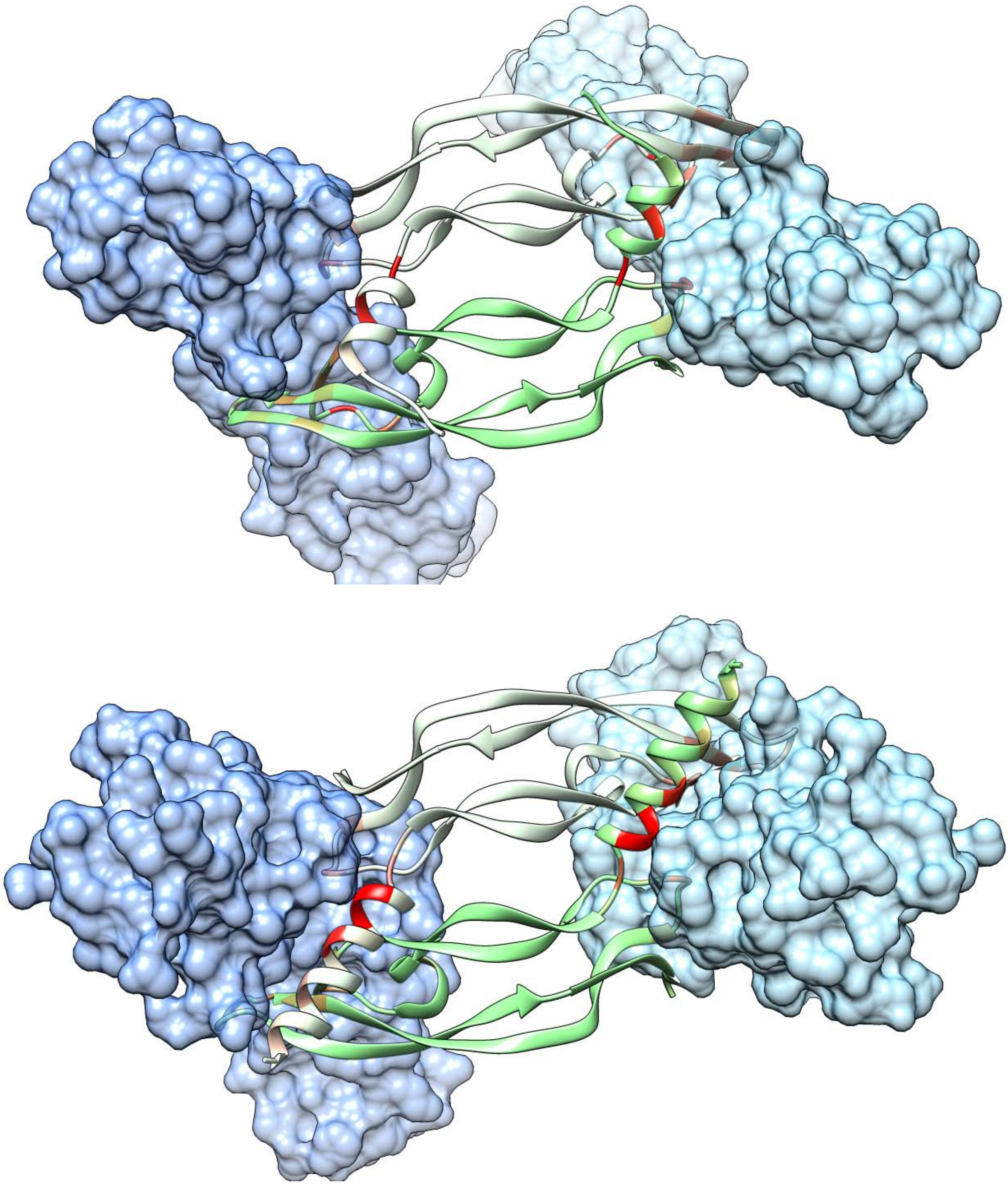

Figure S1: *In silico* Ala scan analysis of the VEGFR-2/VEGF-A (PDB code 3V2A, top) and VEGFR-2/VEGF-C (PDB code 2X1X, bottom) complexes. The receptor Ig-homology domains D2 and D3 are shown in surface representation (light blue and blue for the two subunits), while the two subunits of VEGF are shown as ribbons of two different shades of green. VEGF residues are colored depending on the standard binding free energy penalty associated with their mutation to Ala, as predicted by DrugScorePPI (values from 0 to 1.5 kcal/mol; are reported in shades of color going from green to red, values 1.5 kcal/mol and above, are all reported in red).

MATERIALS AND METHODS

All manipulations requiring anhydrous conditions were carried out in flame-dried glassware, with magnetic stirring and under nitrogen atmosphere. All commercially available reagents were used as received. The resin and the amino acids used in the SPPS were purchased from Merck KGaA Novabiochem®. Dry iPr$_2$NEt was distilled over CaH_2 and transferred under nitrogen. Dry DMF (over molecular sieves in bottles with crown cap) was purchased from Sigma Aldrich, stored under nitrogen, and withdrawn from the container by syringe, under a slight positive pressure of nitrogen. ESI-MS spectra were recorded on the ion trap mass spectrometer Finnigan LCQ Advantage analysing a solution H_2O/CH_3CN 1:1 + 0,1% TFA. MALDI-TOF-MS spectra were recorded on the instrument Bruker Microflex™ LT, supporting the sample on the 2,5-dihydroxybenzoic acid (DHB), α-cyano-4-hydroxycinnamic acid (HCCA) and sinapinic acid (SIN) matrices. The peptide calibration standard for the MALDI-TOF-MS instrument (300-3000 Da range), consisting of Angiotensin II, Angiotensin I, Substance P, Bombesin; ACTH clip 1-17, ACTH clip 18-39, Somatostatin 28, was purchased from Bruker Daltonics®. The sample was mixed in equal volumes with the matrix solution: a small amount (1 µL) of this mixture was spotted on the target surface. The target matrix was dried at room temperature and then analyzed.

HPLC PURIFICATION

The HPLC purifications were performed using a Dionex Ultimate 3000 instrument equipped with a Dionex RS Variable Wavelength Detector (column: Atlantis® Prep T3 OBD™ 5 µm 19 × 100 mm). The crude reaction mixture was dissolved in H_2O or, if the compound was insoluble in water, adding first DMF, then diluting slowly with H_2O until reaching at least a 1:1 mixture DMF/H_2O (ultrasonic sonicator was used to assist the dissolution). The solution so obtained was filtered (polypropylene, 0.45 µm, 13 mm ø, PK/100) and injected in the HPLC, affording purified products.

HPLC ANALYSIS

Purity analysis was carried out on a Dionex Ultimate 3000 instrument equipped with a Dionex RS Variable Wavelenght detector (column: Atlantis® Prep T3 OBD™ 5 µm 19 × 100 mm). 1 mg of analyte was dissolved in 1 mL of H_2O and injected. The analysis was performed using the same gradient used in the purification step. The analysis of the integrals and the relative percentage of purity was performed with the software Cromeleon 6.80 SR11 Build 3161.

FREEZE-DRYING

The product was dissolved in glacial acetic acid and frozen with dry ice: the freeze-drying was carried out at least for 48 h at -50 °C using the instrument 5Pascal Lio5P DGT.

SOLID PHASE RECEPTOR BINDING ASSAYS ON VEGFR-1

The surface of a white high-binding 96-well microplate (Corning Life Sciences, Netherlands) was coated with 100 µL of phosphate-buffered saline solution (PBS, pH 7.4) containing 200 ng/mL of VEGFR-1 ECD/Fc chimera (R& D Systems, Minneapolis, MN, USA) and incubated overnight at 4 °C. After three washes with 150 µL of PBS 0.1%, (v/v) Tween 20 (buffer A), the plate was blocked by 160 µL of PBS with 3% (w/v) of BSA and incubated at r.t. for 2 h. The plate was washed three times with buffer A. Then, 100 µL of a solution of btVEGF$_{165}$ at 131 pm (5 ng/mL) and the tested compounds at various concentrations diluted in PBS containing 5% DMSO were added to each well. After 3 h at 37 °C, the plate was washed three times with buffer A and 100 µL of streptavidin–

horseradish peroxidase diluted at 1:1000 in PBS containing 0.1% (v/v) Tween 20 and 0.3% (w/v) BSA were added per well. After 1 h of incubation at r.t., the plate was washed five times with 150 µL of buffer A and 100 µL of SuperSignal West Pico Chemiluminescent Substrate (Pierce, Rockford, IL, USA) were added. The remaining bt-$VEGF_{165}$ was detected by chemiluminescence, which was quantified with a Synergy™ HT Multi-Detection Microplate Reader (BioTek Instruments, Inc.). The percentages of bt$VEGF_{165}$ displacement were calculated by the following formula: percentage of displacement = $100\times[1-(S-NS) / (MS-NS)]$, where S is the signal measured, NS is the nonspecific binding signal and MS is the maximum binding signal observed with bt$VEGF_{165}$ without tested compounds.

CELL CULTURES

HUVEC were cultured in a medium supplemented with FBS (2%), l-glutamine (10 mM), heparin sulfate (0.75 U/mL), VEGF (15 ng/mL), EGF (5 ng/mL), FGF2 (5 ng/mL), IGF-I (15 ng/mL) and ascorbic acid (50 µg/mL) at 37 °C, in a moist atmosphere of 5% CO_2. HUVEC were used for the experiment between passage 2 to 10.

STATISTICAL ANALYSIS OF IN VITRO MORPHOGENESIS ASSAYS ON HUVEC

Data are shown as means ± standard deviation (SD) unless otherwise indicated. Statistical significance of the differences was assessed by two-tailed Student's *t* test for paired data. Calculations were performed using a commercial software (GraphPad Prism version 5.00 for Windows, GrapPad Software, San Diego California USA, www.graphpad.com).

GENERAL PROCEDURES FOR SEMI-AUTOMATIC SPPS

The semi-automatic SPPS was accomplished through the Biotage® Initiator™ synthesizer, assisted by microwave (MW) irradiation; Fmoc/*t*Bu strategy and Rink Amide MHBA Resin (100-200 mesh; loading: 0.5 mmol/g) were used.
Each coupling step consisted in:

1) activation of the Fmoc-protected amino acid;
2) addition of the activated amino acid to the resin at the synthesizer to start the coupling;
3) steps of capping, deprotection and washing.

SOLUTIONS AND SOLVENTS

To perform the automated SPPS, two solutions and two solvents were prepared: 25% Ac_2O in DMF (v/v), 25% piperidine in DMF (v/v), methanol and DMF. While MeOH was used only as washing solvent, DMF was necessary either for washings and as solvent for the reactions of coupling, capping and deprotection.

RESIN PREPARATION

The resin was weighted in a 10 mL Teflon vial (Biotage) and processed with the swelling task and the swelling-Fmoc deprotection task. At the end of these operations the resin was ready for the SPPS.

ACTIVATION OF FMOC-AA-OH

To a solution of the desired Fmoc-AA-OH (4.0 eq with respect to the resin) in DMF (3.5 mL, dry solvent) DIC (4.0 eq), HOAt (4.0 eq) and *i*Pr_2NEt (8.0 eq) were added at 0 °C, under stirring and inert atmosphere. After 25 min, the reaction mixture was added to the resin in the reaction vessel of the synthesizer.

BIOTAGE® INITIATOR™ PROGRAMS

The Biotage® Initiator™ programs ("Tasks") used for the semi-automatic SPPS are reported below. Each task can be modified in every parameter and it is performed under vortex mixing at 1000 rpm.

Swelling task: DMF (3.0 mL) was added to the resin: the swelling was accomplished at r.t. in 30 min. At the end of the swelling step, the liquid phase was drained.

Swelling-Fmoc deprotection task: DMF (3.5 mL) was added to the resin and the swelling step was performed at r.t. for 15 min. Two deprotection steps were carried out adding 25% piperidine in DMF (3.0 mL for each step): the reaction was performed at r.t. under inert atmosphere for 5 min and 15 min for the first and the second deprotection step, respectively. The resin was washed eight times with DMF (3 mL × 30 s for every wash). At the end of each step, the liquid phase was drained.

Coupling (Double coupling)-capping-Fmoc deprotection: The activated Fmoc-AA-OH residue was added to the resin in the reaction vessel of the synthesizer and the coupling reaction assisted by microwaves was carried out at 70 °C under inert atmosphere for 12 min. When a double coupling was required, another aliquot of the activated Fmoc-AA-OH residue was added to the resin and a second coupling reaction was performed. The beads were washed six times with DMF (2.5 mL × 20 s for every wash). The capping solution (3 mL) was added to the resin: the capping step was performed at r.t. under inert atmosphere for 15 min. The beads were washed six times with DMF (2.5 mL × 20 s for every wash). Two deprotection steps were carried out adding the deprotection solution (3.0 mL for each step) to the beads: the reaction was performed at r.t. under inert atmosphere for 5 min and 7 min for the first and the second deprotection step, respectively. The beads were washed twice with DMF, MeOH, DMF, MeOH and DMF (3.0 mL × 20 s for every wash; 3.0 mL × 20 s for the last washes in MeOH and DMF). At the end of each step, the liquid phase was drained.

Coupling (Double coupling)-capping: The activated Fmoc-AA-OH residue was added to the resin in the reaction vessel of the synthesizer, and the coupling reaction assisted by microwaves was carried out at 70 °C under inert atmosphere for 12 min. When a double coupling was required, another aliquot of the activated Fmoc-AA-OH was added to the resin and a second coupling reaction was performed. The beads were washed six times with DMF (2.5 mL × 20 s for every wash). The capping solution (3 mL) was added to the resin: the capping step was performed at r.t. under inert atmosphere for 15 min. The beads were washed six times with DMF (2.5 mL × 20 s for every wash). The beads were washed twice with DMF, MeOH, DMF, MeOH (3.0 mL × 20 s for every wash). Eventually, the beads were washed eight times with DMF (3.0 mL × 30 s). At the end of each step, the liquid phase was drained.

Capping: The solution 25% Ac_2O in DMF (3 mL) was added to the resin: the capping step was performed at r.t. under inert atmosphere for 15 min. The beads were washed six times with DMF (2.5 mL × 20 s for every wash). The beads were washed twice with DMF, MeOH, DMF (3.0 mL ×

20 s for every wash). Eventually, the beads were washed twice with MeOH (3.0 mL × 30 s) and eight times with DMF (3.0 mL × 30 s). At the end of each step, the liquid phase was drained.

RESIN STORAGE

The on-beads peptides were stored at -20 °C in a small volume of DMF (2-3 mL), with the terminal amino group of the peptide left Fmoc-protected. The next SPPS cycle restarted with a swelling-deprotection step before continuing the peptide synthesis.

GENERAL PROCEDURE FOR KAISER TEST ON SOLID PHASE

A few drops of solution A (80% phenol solution in ethanol), solution B (6% ninhydrin solution in ethanol) and solution C (98:2 pyridine/KCN aq. 0.1 mM) were added to a small sample of the resin and then shaken at 100 °C for 1 min. If the solution maintained its yellow color, quantitative coupling was achieved. On the contrary, in case of a slight blue color, the coupling step was not fully completed and it was then repeated.

PEPTIDE CLEAVAGE

All the cleavage reactions from the resin were performed manually, under inert atmosphere and vortex mixing. The protected on-beads peptide was swollen first with DMF (3.5 mL), then with dichloromethane (3.5 mL). Under stirring and nitrogen atmosphere, the beads were treated three times with the cleavage cocktail (3.0 mL per 0.1 mmol of resin) 90:5:3:2 TFA / thioanisole / EDT / anisole (v/v/v/v). After 1 h, the liquid phase was filtered off under nitrogen flow and collected in a round bottom flask: the beads were washed with neat TFA (1.0 mL) that was collected. The combined filtered fractions were concentrated and poured in cold diethyl ether, provoking precipitation of the product. Diethyl ether was removed with a syringe, affording the crude product, which was purified by RP-HPLC and freeze-dried.

SYNTHESIS OF VEGF-C DERIVED PEPTIDES 1-6

The synthesis of VEGF-C derived peptide sequences (**1**-**6**, Figure S1) was accomplished as described in the General Procedures for Solid-Phase Synthesis on Rink Amide MHBA Resin (200 mg, 0.100 mmol).

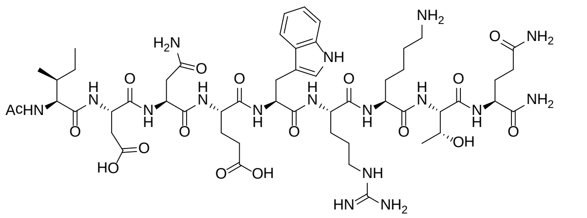

1
Ac-Ile-Asp-Asn-Glu-Trp-Arg-Lys-Thr-Gln-amide
(natural VEGF-C fragment)

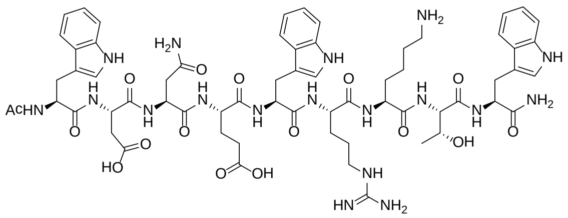

2
Ac-Trp-Asp-Asn-Glu-Trp-Arg-Lys-Thr-Trp-amide

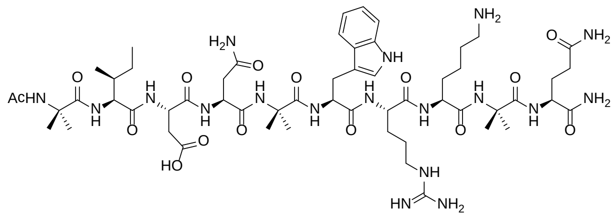

3
Ac-Aib-Ile-Asp-Asn-Aib-Trp-Arg-Lys-Aib-Gln-amide

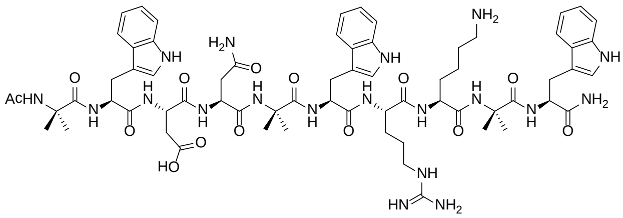

4
Ac-Aib-Trp-Asp-Asn-Aib-Trp-Arg-Lys-Aib-Trp-amide

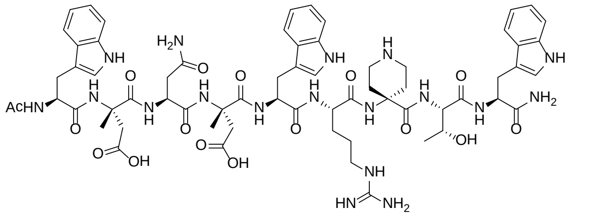

5
Ac-Trp-(αMe)Asp-Asn-(αMe)Asp-Trp-Arg-Api-Thr-Trp-amide

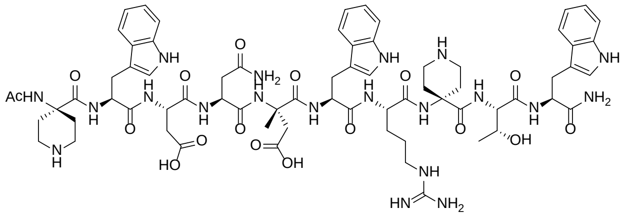

6
Ac-Api-Trp-Asp-Asn-(αMe)Asp-Trp-Arg-Api-Thr-Trp-amide

Figure S2. VEGF-C derived peptides **1**-**6**.

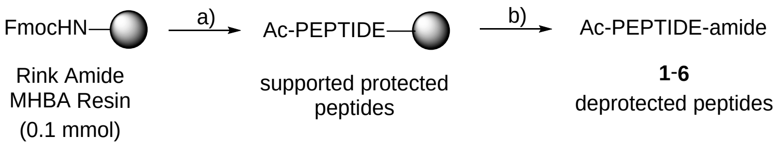

Scheme S1. Reagents and conditions: a) SPPS: 1. 25% piperidine in DMF; 2. Fmoc-AA-OH (4.0 eq), DIC, HOAt, *i*Pr_2NEt, DMF; 3. 25% Ac_2O in DMF; b) TFA/thioanisole/EDT/anisole 90:5:3:2 v/v/v/v, r.t., 3 h, 7-30%.

The exact amounts of the amino acids used for each peptide sequence are reported in the corresponding Table.

Natural VEGF-C fragment Ac-Ile-Asp-Asn-Glu-Trp-Arg-Lys-Thr-Gln-amide (**1**)

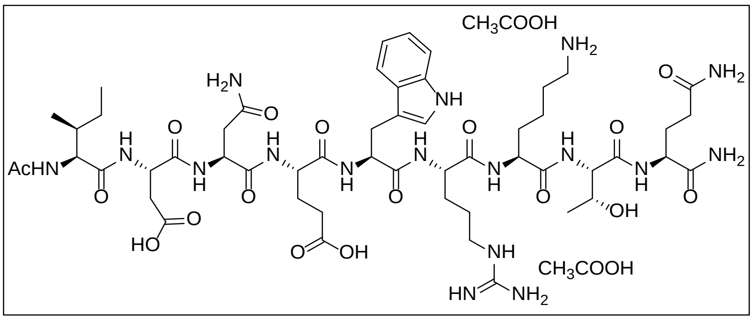

Chemical Formula: $C_{57}H_{91}N_{17}O_{21}$
Molecular Weight: 1350.45

Table S1. Amounts of amino acid derivatives used in the SPPS of compound **1**.

Fmoc-AA-OH	Molecular Weight (g/mol)	Amount (mg)
Fmoc-Gln(Trt)-OH	610.70	244
Fmoc-Thr(*t*Bu)-OH	397.46	159
Fmoc-Lys(Boc)-OH	468.54	187
Fmoc-Arg(Pbf)-OH	648.78	260
Fmoc-Trp(Boc)-OH	526.58	211
Fmoc-Glu(O*t*Bu)-OH	425.47	170
Fmoc-Asn(Trt)-OH	596.69	239
Fmoc-Asp(O*t*Bu)-OH	411.46	165
Fmoc-Ile-OH	353.41	141

The supported peptide (0.100 mmol) was fully deprotected and cleaved from the resin as described in the general procedures and purified by RP-HPLC ($t_{R\ (product)}$ = 7.9 min).

The desired peptide **1** was freeze-dried, obtaining the corresponding salt as white solid (6 mg, 12%).

MS (MALDI-TOF) *m/z* calcd. for $[C_{53}H_{84}N_{17}O_{17}]^+$: 1231.6; found:1231.2 $[M+H]^+$ (SIN matrix).

Peptide Ac-Trp-Asp-Asn-Glu-Trp-Arg-Lys-Thr-Trp-amide (**2**)

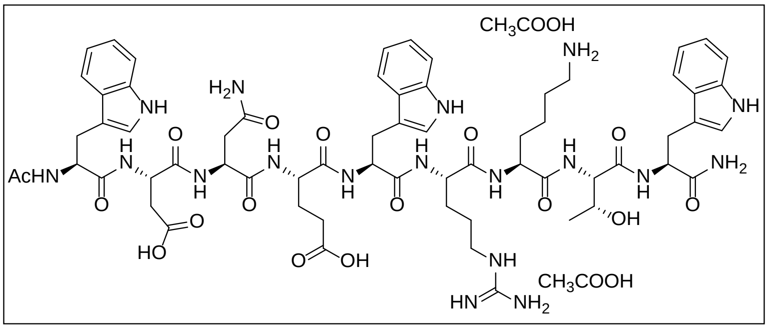

Table S2. Amounts of amino acid derivatives used in the SPPS of compound **2**.

Fmoc-AA-OH	Molecular Weight (g/mol)	Amount (mg)
Fmoc-Trp(Boc)-OH	526.58	211
Fmoc-Thr(*t*Bu)-OH	397.46	159
Fmoc-Lys(Boc)-OH	468.54	187
Fmoc-Arg(Pbf)-OH	648.78	260
Fmoc-Trp(Boc)-OH	526.58	211
Fmoc-Glu(O*t*Bu)-OH	425.47	170
Fmoc-Asn(Trt)-OH	596.69	239
Fmoc-Asp(O*t*Bu)-OH	411.46	165
Fmoc-Trp(Boc)-OH	526.58	211

The supported peptide (0.100 mmol) was fully deprotected and cleaved from the resin as described in the general procedures and purified by RP-HPLC ($t_{R\ (product)}$ = 11.2 min).

The desired peptide **2** was freeze-dried, obtaining the corresponding salt as white solid (22 mg, 16%).

MS (MALDI-TOF) *m/z* calcd. for $[C_{64}H_{85}N_{18}O_{16}]^+$: 1361.6; found: 1361.6 $[M+H]^+$ (HCCA matrix).

Peptide Ac-Aib-Ile-Asp-Asn-Aib-Trp-Arg-Lys-Aib-Gln-amide (**3**)

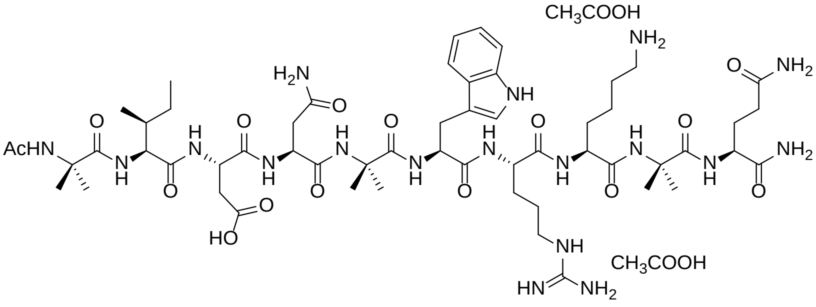

Chemical Formula: $C_{60}H_{98}N_{18}O_{19}$
Molecular Weight: 1375.55

Table S3. Amounts of amino acid derivatives used in the SPPS of compound **3**.

Fmoc-AA-OH	Molecular Weight (g/mol)	Amount (mg)
Fmoc-Gln(Trt)-OH	610.70	244
Fmoc-Aib-OH*	325.36	130
		130
Fmoc-Lys(Boc)-OH	468.54	187
Fmoc-Arg(Pbf)-OH	648.78	260
Fmoc-Trp(Boc)-OH	526.58	211
Fmoc-Aib-OH*	325.36	130
		130
Fmoc-Asn(Trt)-OH	596.69	239
Fmoc-Asp(O*t*Bu)-OH	411.46	165
Fmoc-Ile-OH	353.41	141
Fmoc-Aib-OH*	325.36	130
		130

*Aib, α-aminoisobutyric acid. A double coupling was performed.

The supported peptide (0.100 mmol) was fully deprotected and cleaved from the resin as described in the general procedures and purified by RP-HPLC ($t_{R\ (product)}$ = 10.6 min).

The desired peptide **3** was freeze-dried, obtaining the corresponding salt as white solid (25 mg, 20%).

MS (MALDI-TOF) *m/z* calcd. for $[C_{56}H_{91}N_{18}O_{15}]^+$: 1256.5; found: 1256.4 $[M+H]^+$ (SIN matrix).

Peptide Ac-Aib-Trp-Asp-Asn-Aib-Trp-Arg-Lys-Aib-Trp-amide (**4**)

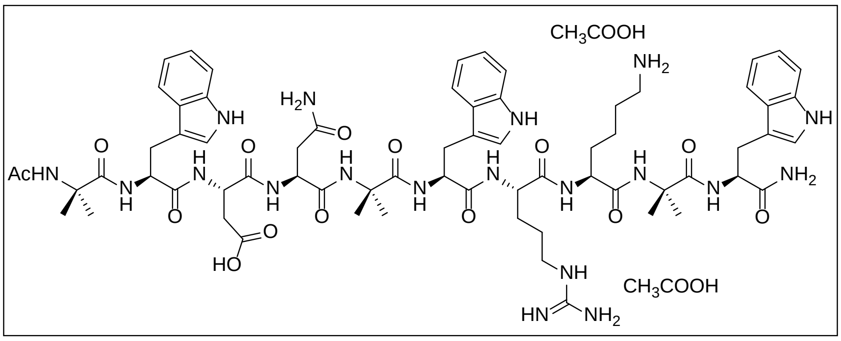

Chemical Formula: $C_{71}H_{99}N_{19}O_{18}$
Molecular Weight: 1506.69

Table S4. Amounts of amino acid derivatives used in the SPPS of compound **4**.

Fmoc-AA-OH	Molecular Weight (g/mol)	Amount (mg)
Fmoc-Trp(Boc)-OH	526.58	211
Fmoc-2-Aminoisobutyric acid-OH*	325.36	130
		130
Fmoc-Lys(Boc)-OH	468.54	187
Fmoc-Arg(Pbf)-OH	648.78	260
Fmoc-Trp(Boc)-OH	526.58	211
Fmoc-2-Aminoisobutyric acid-OH*	325.36	130
		130
Fmoc-Asn(Trt)-OH	596.69	239
Fmoc-Asp(O*t*Bu)-OH	411.46	165
Fmoc-Trp(Boc)-OH	526.58	211
Fmoc-2-Aminoisobutyric acid-OH*	325.36	130
		130

*A double coupling was performed.

The supported peptide (0.100 mmol) was fully deprotected and cleaved from the resin as described in the general procedures and purified by RP-HPLC ($t_{R\ (product)}$ = 12.6 min).

The desired peptide **4** was freeze-dried, obtaining the corresponding acetate salt as white solid (41 mg, 30%).

MS (MALDI-TOF) *m/z* calcd. for $[C_{67}H_{92}N_{19}O_{14}]^+$: 1387.6; found: 1387.3 $[M+H]^+$ (SIN matrix).

Peptide Ac-Trp-(αMe)Asp-Asn-(αMe)Asp-Trp-Arg-Api-Thr-Trp-amide (**5**)

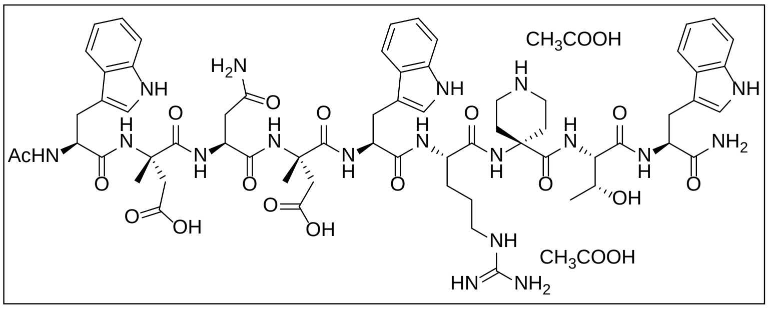

Table S5. Amounts of amino acid derivatives used in the SPPS of compound **5**.

Fmoc-AA-OH	Molecular Weight (g/mol)	Amount (mg)
Fmoc-Trp(Boc)-OH	526.58	211
Fmoc-Thr(*t*Bu)-OH	397.46	159
Fmoc-Api(Boc)-OH*	466.53	187
		187
Fmoc-Arg(Pbf)-OH*	648.78	260
		260
Fmoc-Trp(Boc)-OH	526.58	211
Fmoc-(αMe)Asp(O*t*Bu)-OH*	425.47	170
		170
Fmoc-Asn(Trt)-OH*	596.69	239
		239
Fmoc-(αMe)Asp(O*t*Bu)-OH*	425.47	170
		170
Fmoc-Trp(Boc)-OH*	526.58	211
		211

*A double coupling was performed.

The supported peptide (0.100 mmol) was fully deprotected and cleaved from the resin as described in the general procedures and purified by RP-HPLC ($t_{R\ (product)}$ = 13.1 min).

The desired peptide **5** was freeze-dried, obtaining the corresponding salt as white solid (18 mg, 13%).

MS (MALDI-TOF) *m/z* calcd. for $[C_{65}H_{85}N_{18}O_{16}]^+$: 1373.6; found: 1373.8 $[M+H]^+$ (HCCA matrix).

Peptide Ac-Api-Trp-Asp-Asn-(αMe)Asp-Trp-Arg-Api-Thr-Trp-amide (**6**)

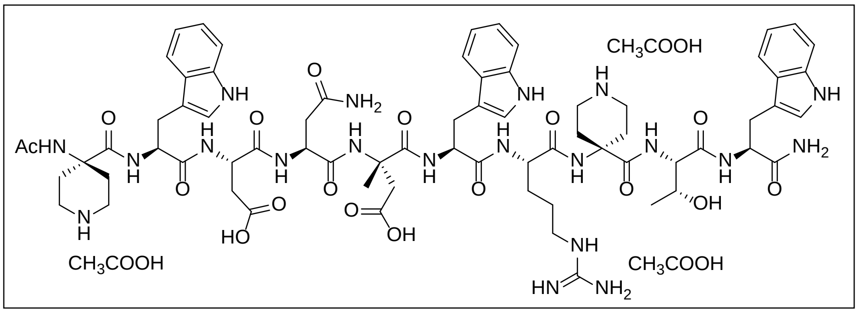

Chemical Formula: $C_{76}H_{104}N_{20}O_{23}$
Molecular Weight: 1665.79

Table S6. Amounts of amino acid derivatives used in the SPPS of compound **6**.

Fmoc-AA-OH	Molecular Weight (g/mol)	Amount (mg)
Fmoc-Trp(Boc)-OH	526.58	211
Fmoc-Thr(*t*Bu)-OH	397.46	159
Fmoc-Api(Boc)-OH*	466.53	187
		187
Fmoc-Arg(Pbf)-OH	648.78	260
Fmoc-Trp(Boc)-OH	526.58	211
Fmoc-(αMe)Asp(O*t*Bu)-OH*	425.47	170
		170
Fmoc-Asn(Trt)-OH	596.69	239
Fmoc-Asp(O*t*Bu)-OH	411.46	165
Fmoc-Trp(Boc)-OH	526.58	211
Fmoc-Api(Boc)-OH*	466.53	187
		187

*A double coupling was performed.

The supported peptide (0.100 mmol) was fully deprotected and cleaved from the resin as described in the general procedures and purified by RP-HPLC ($t_{R\ (product)}$ = 11.7 min).

The desired peptide **6** was freeze-dried, obtaining the corresponding acetate salt as white solid (11 mg, 7%).

MS (MALDI-TOF) *m/z* calcd. for $[C_{70}H_{93}N_{20}O_{17}]^+$: 1485.7; found: 1485.7 $[M+H]^+$ (HCCA matrix).

HPLC CHROMATOGRAMS

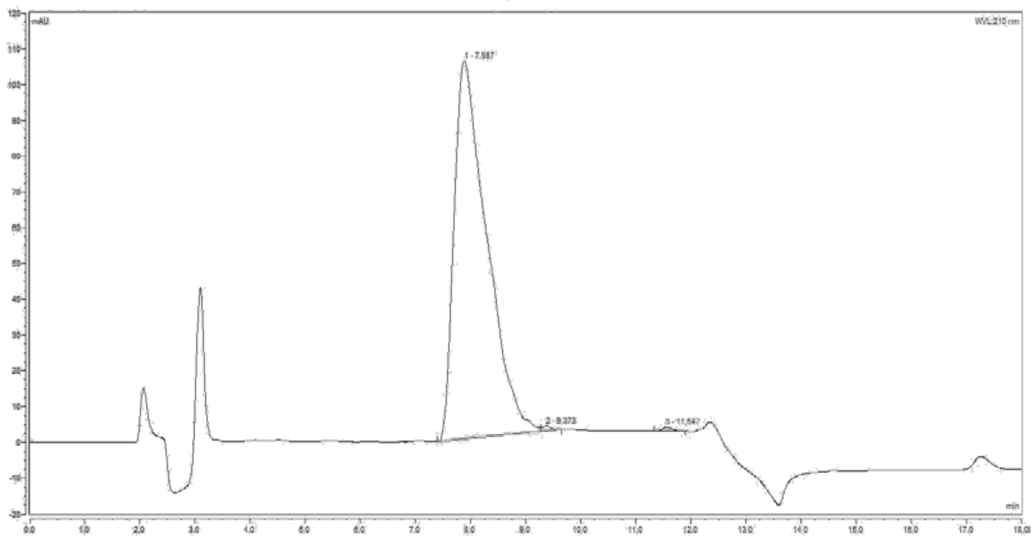

Figure S3: Ac-Ile-Asp-Asn-Glu-Trp-Arg-Lys-Thr-Gln-amide (**1**). Purity: >99%

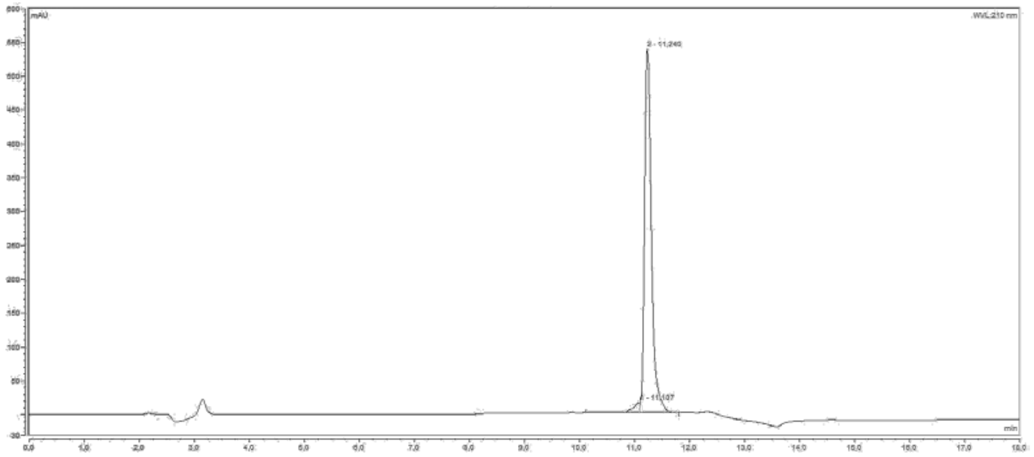

Figure S4: Ac-Trp-Asp-Asn-Glu-Trp-Arg-Lys-Thr-Trp-amide (**2**). Purity: 97%

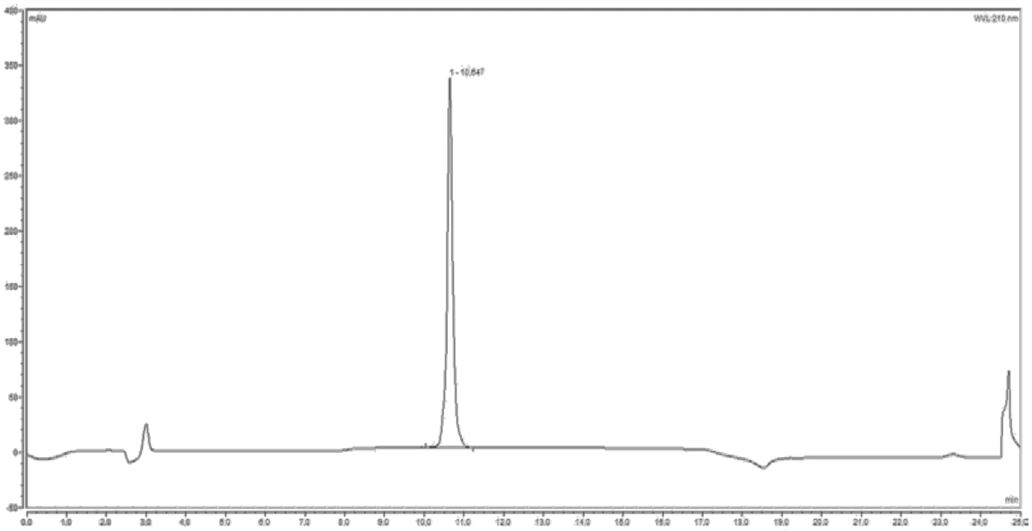

Figure S5: Ac-Aib-Ile-Asp-Asn-Aib-Trp-Arg-Lys-Aib-Gln-amide (**3**). Purity: >99%

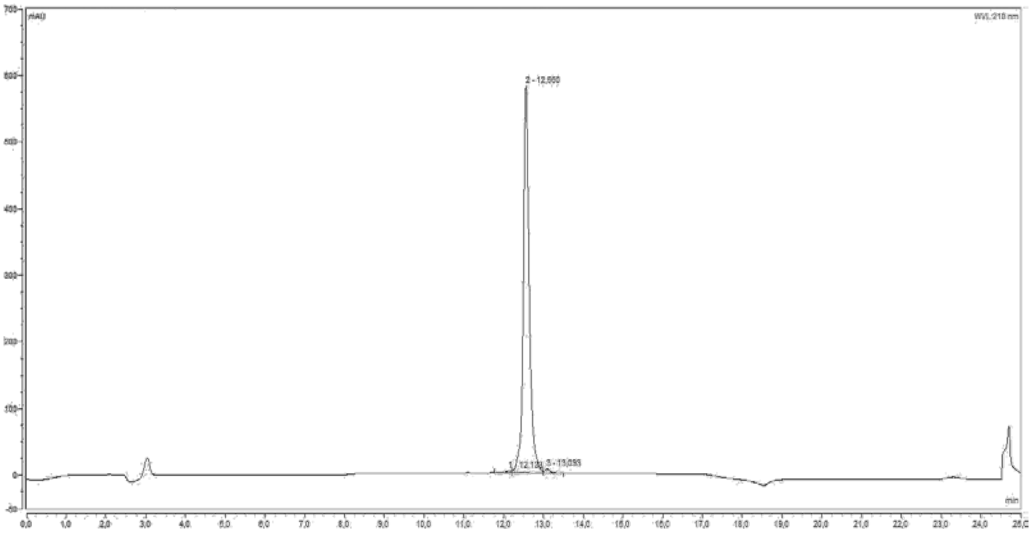

Figure S6: Ac-Aib-Trp-Asp-Asn-Aib-Trp-Arg-Lys-Aib-Trp-amide (**4**). Purity: 99%

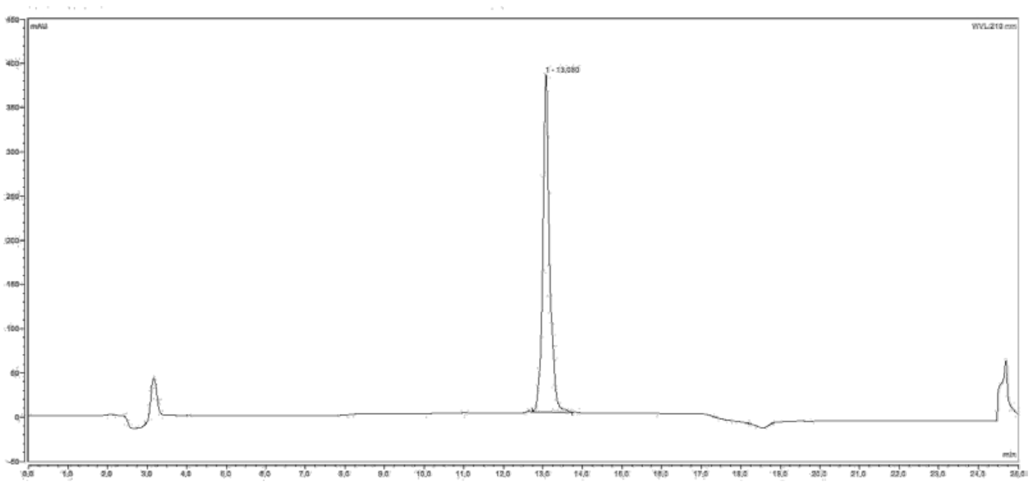

Figure S7: Ac-Trp-(αMe)Asp-Asn-(αMe)Asp-Trp-Arg-Api-Thr-Trp-amide (**5**). Purity: >99%

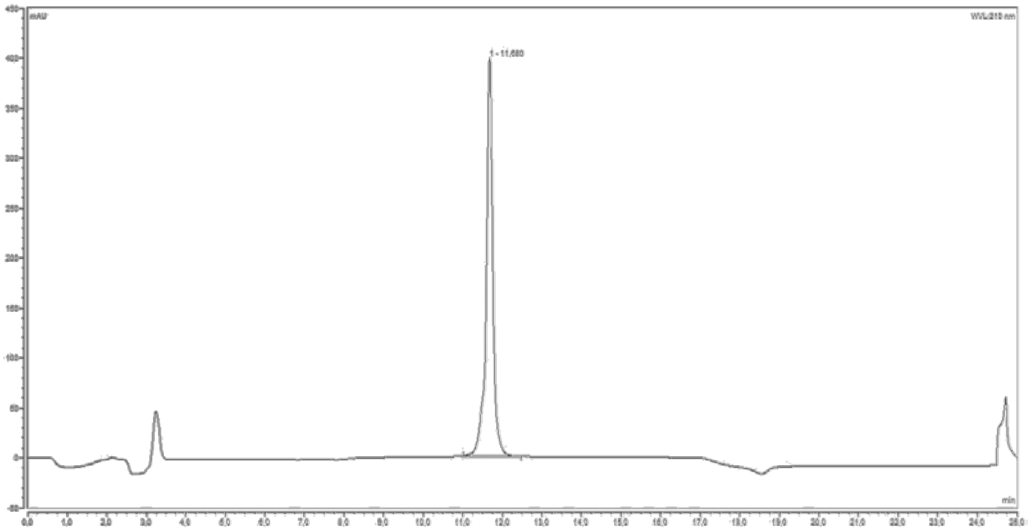

Figure S8: Ac-Api-Trp-Asp-Asn-(αMe)Asp-Trp-Arg-Api-Thr-Trp-amide (**6**). Purity: >99%

MS SPECTRA

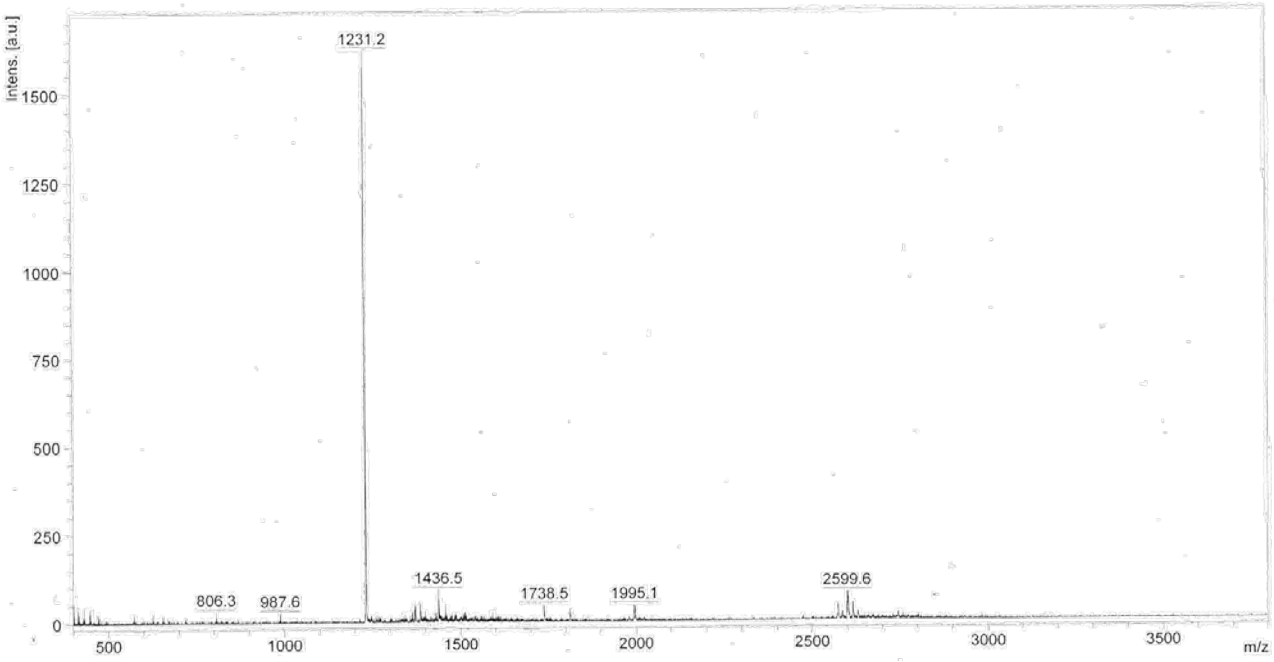

Figure S9: peptide **1**. MALDI-MS (SIN matrix)

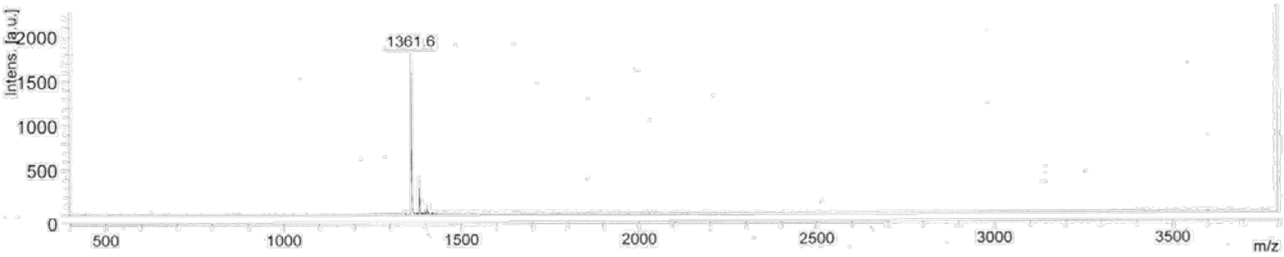

Figure S10: peptide **2**. MALDI-MS (HCCA matrix)

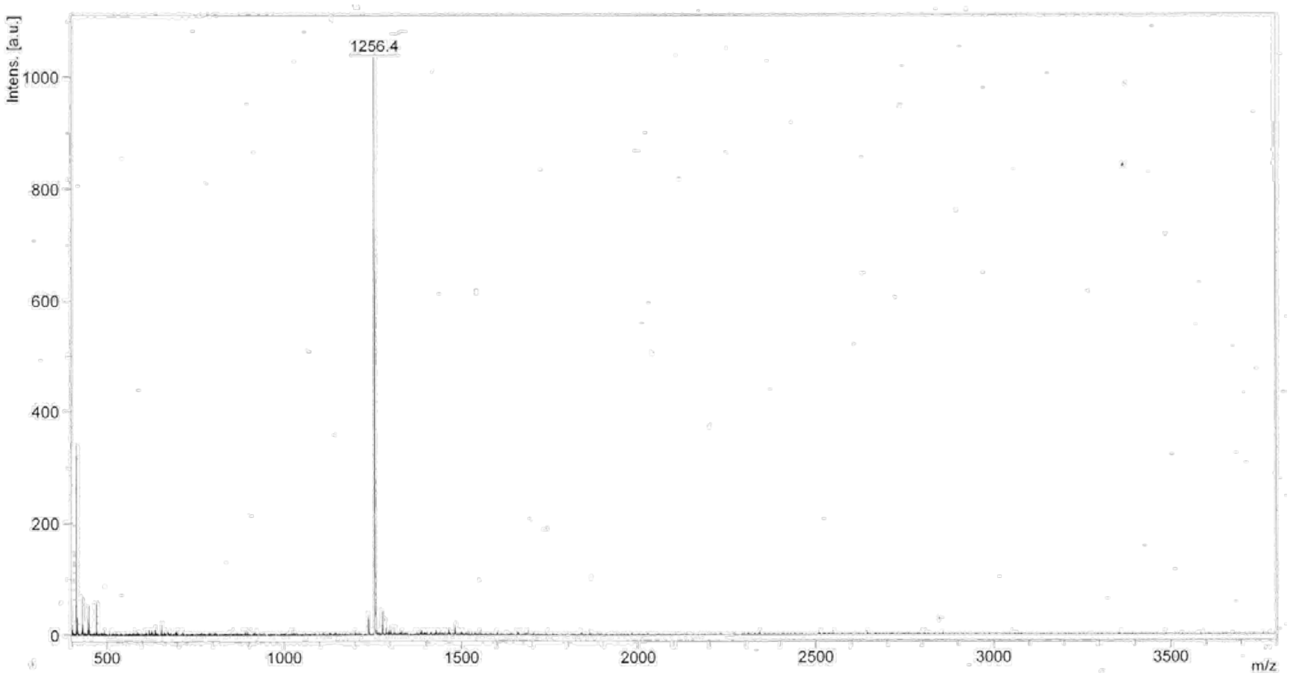

Figure S11: peptide **3**. MALDI-MS (SIN matrix)

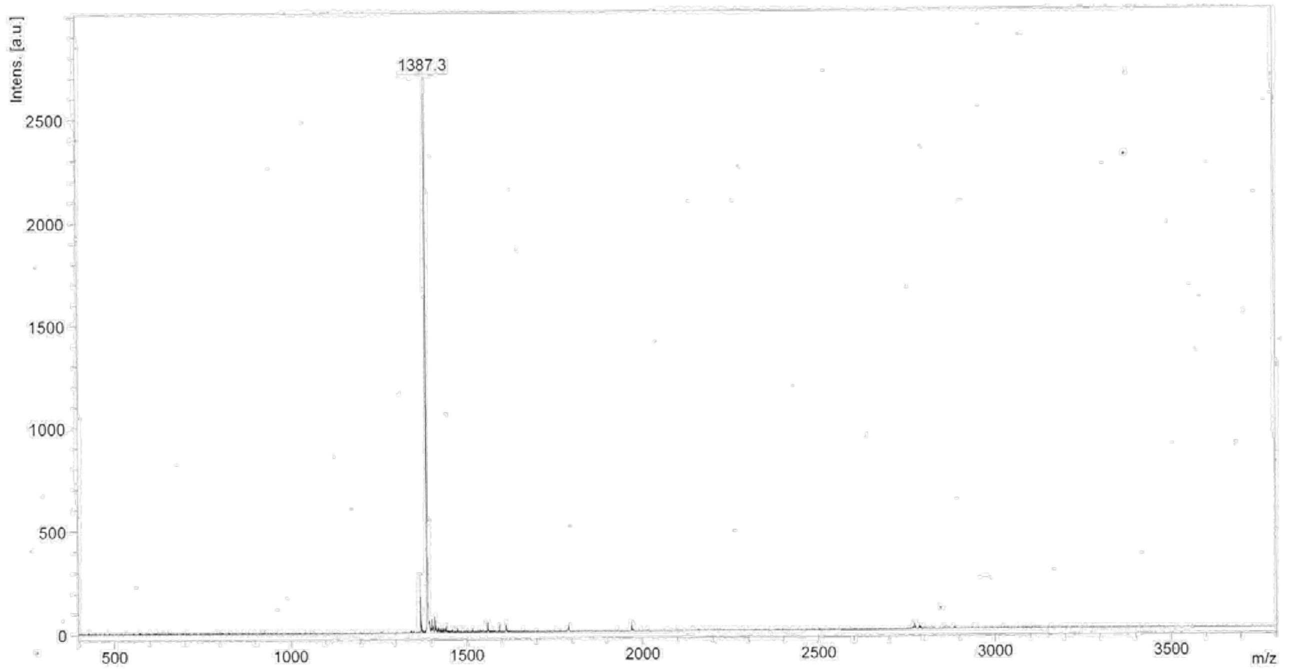

Figure S12: peptide **4**. MALDI-MS (SIN matrix)

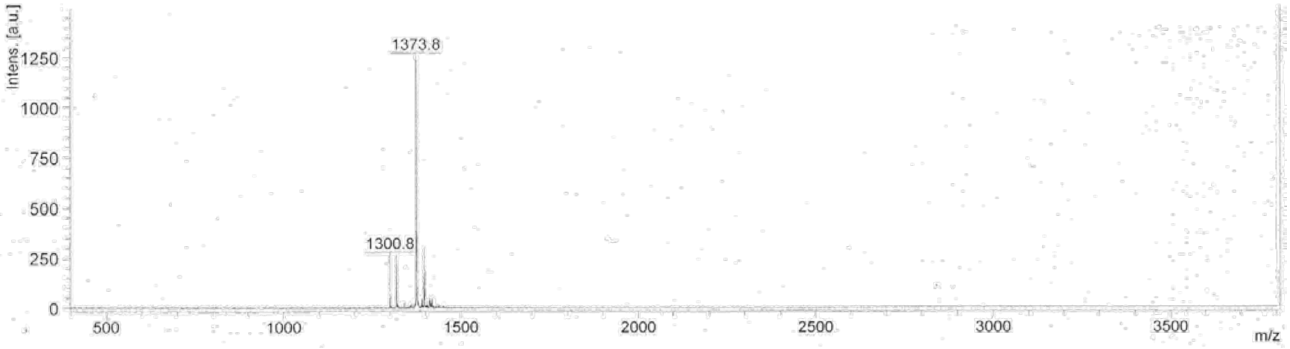

Figure S13: peptide **5**. MALDI-MS (HCCA matrix)

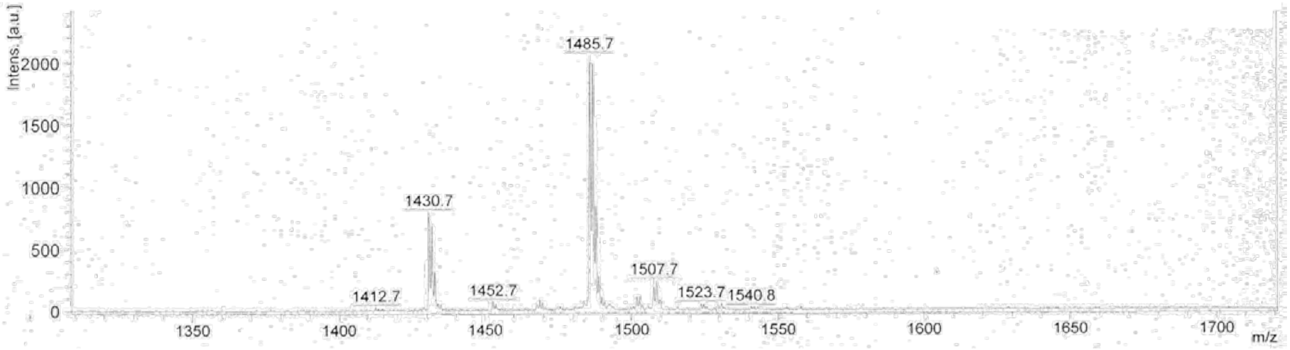

Figure S14: peptide **6**. MALDI-MS (HCCA matrix)

ENZYMATIC DEGRADATION

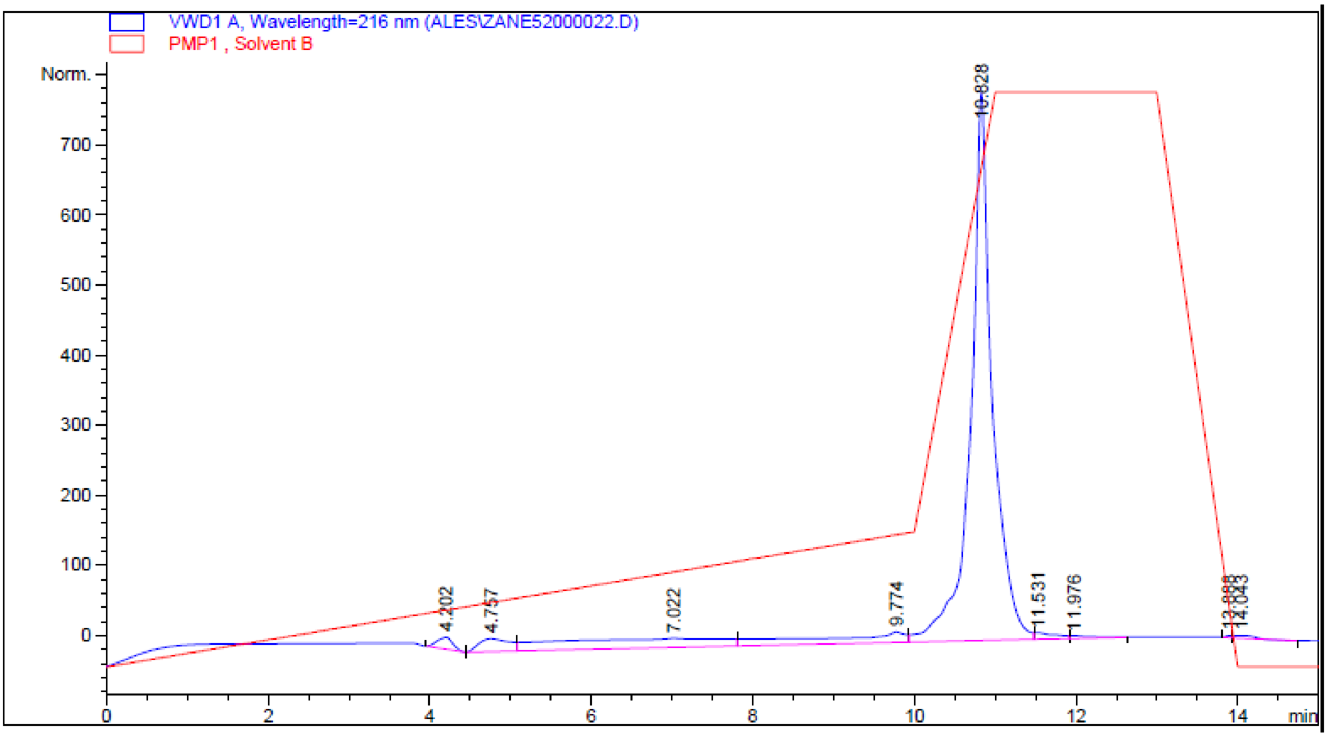

Figure S15. HPLC profile of peptide **5** after 6 days in buffer. (control)

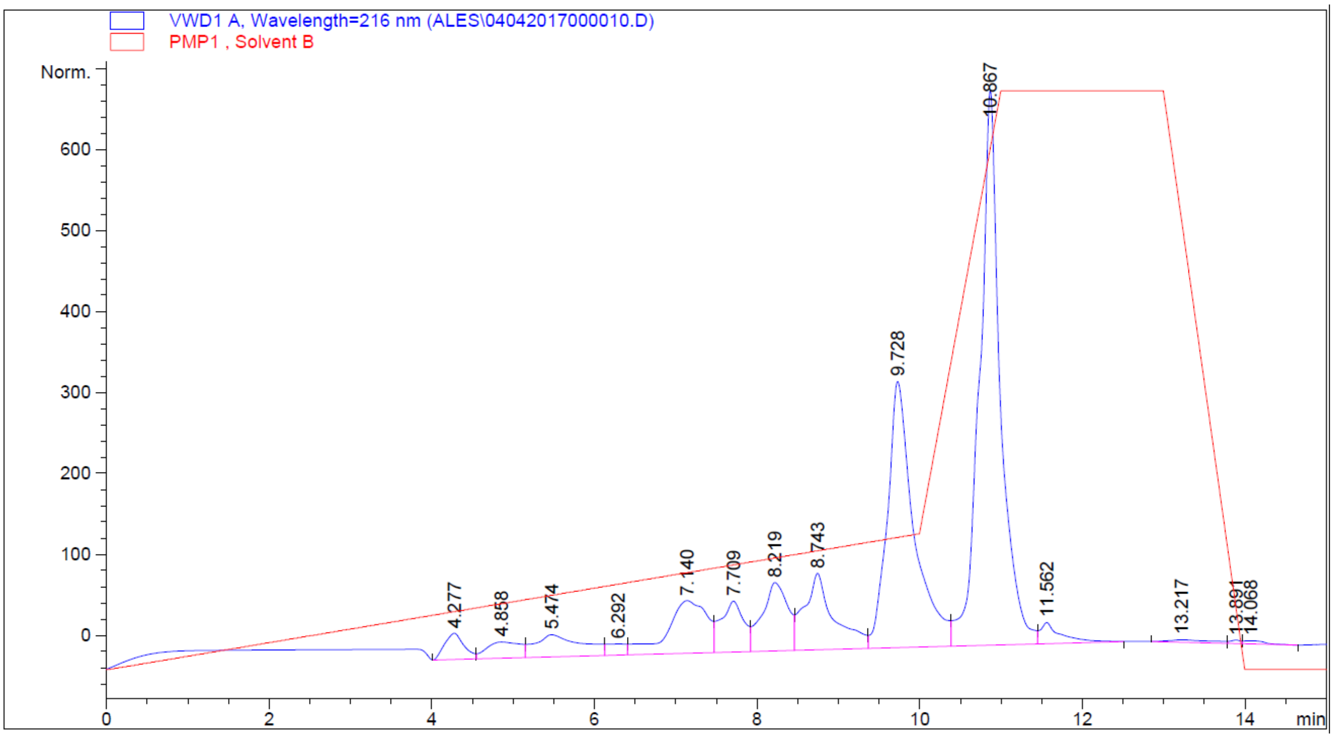

Figure S16. HPLC profile of peptide **5** after 13 days in the presence of pronase.

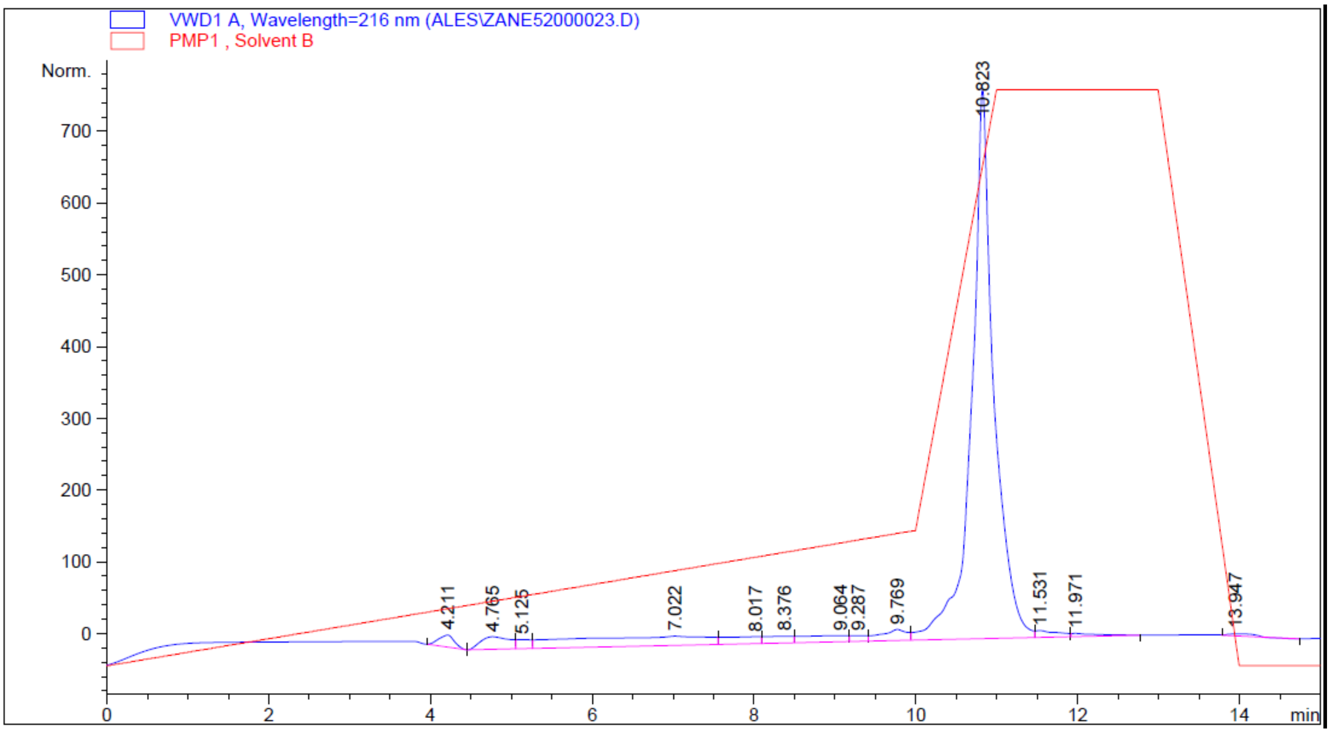

Figure S17. HPLC profile of peptide **5** after 90 min in trypsin.

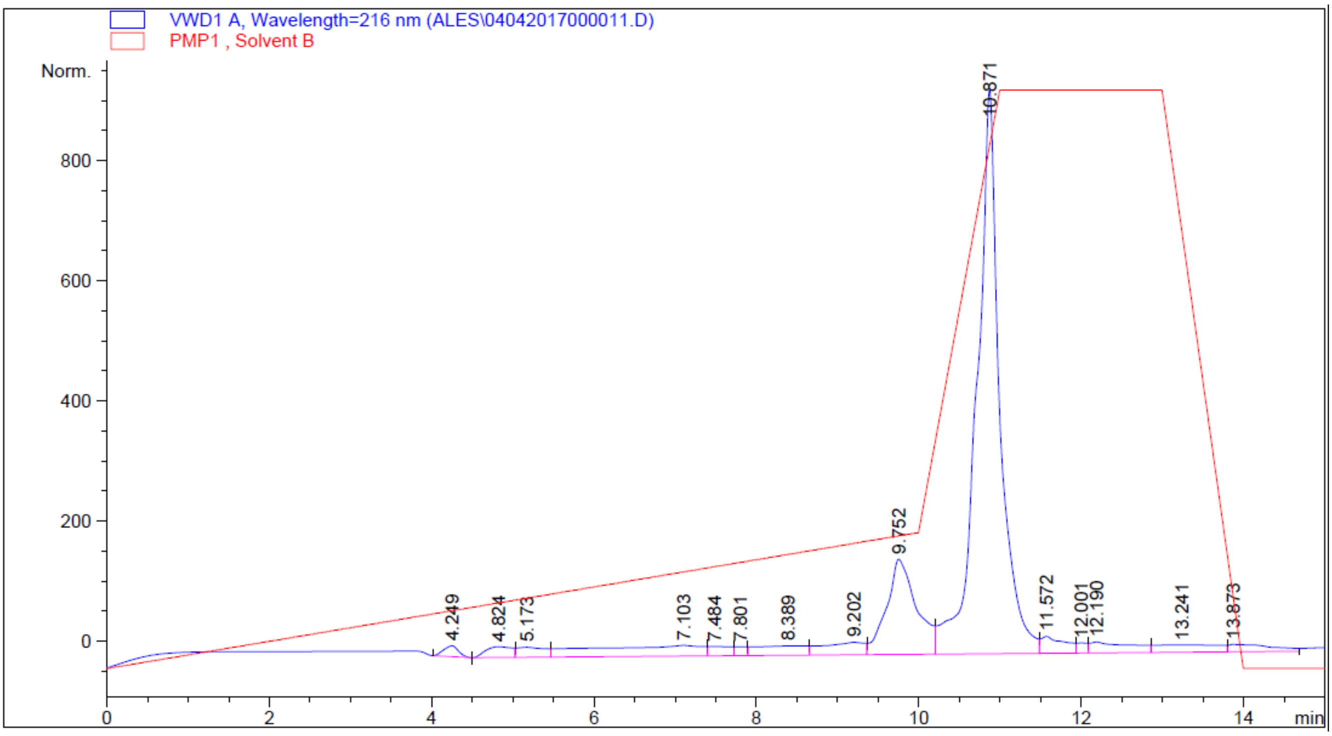

Figure S18. HPLC profile of peptide **5** after six days in trypsin.

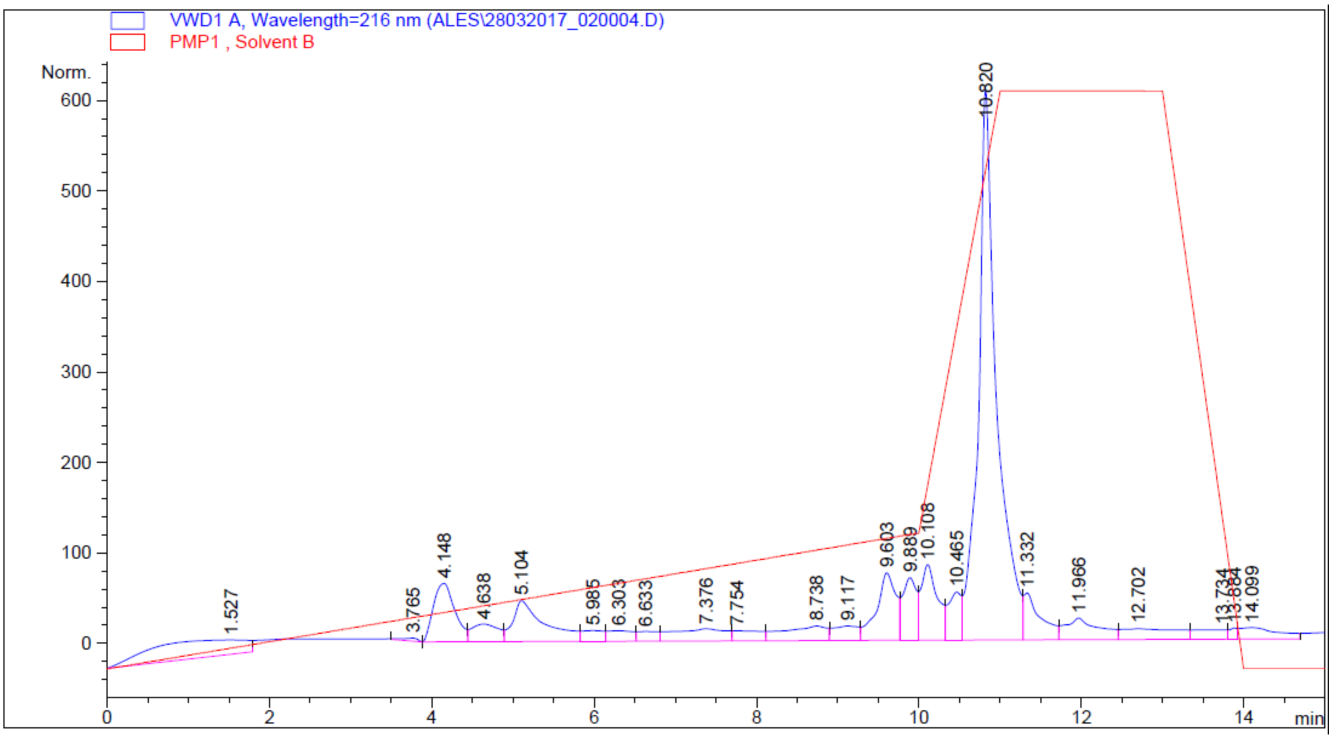

Figure S19. HPLC profile of peptide **5** after four days in chymotrypsin.

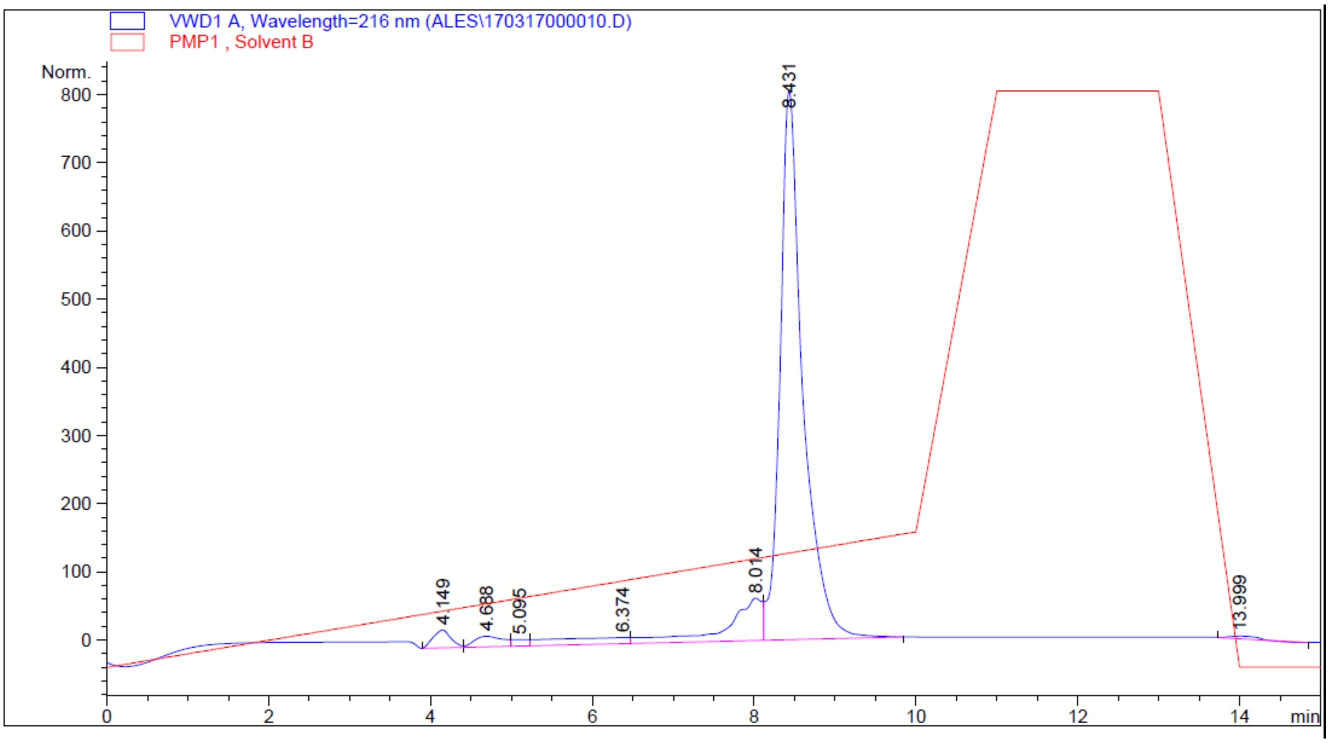

Figure S20: HPLC profile of peptide **6** after 6 days in buffer.

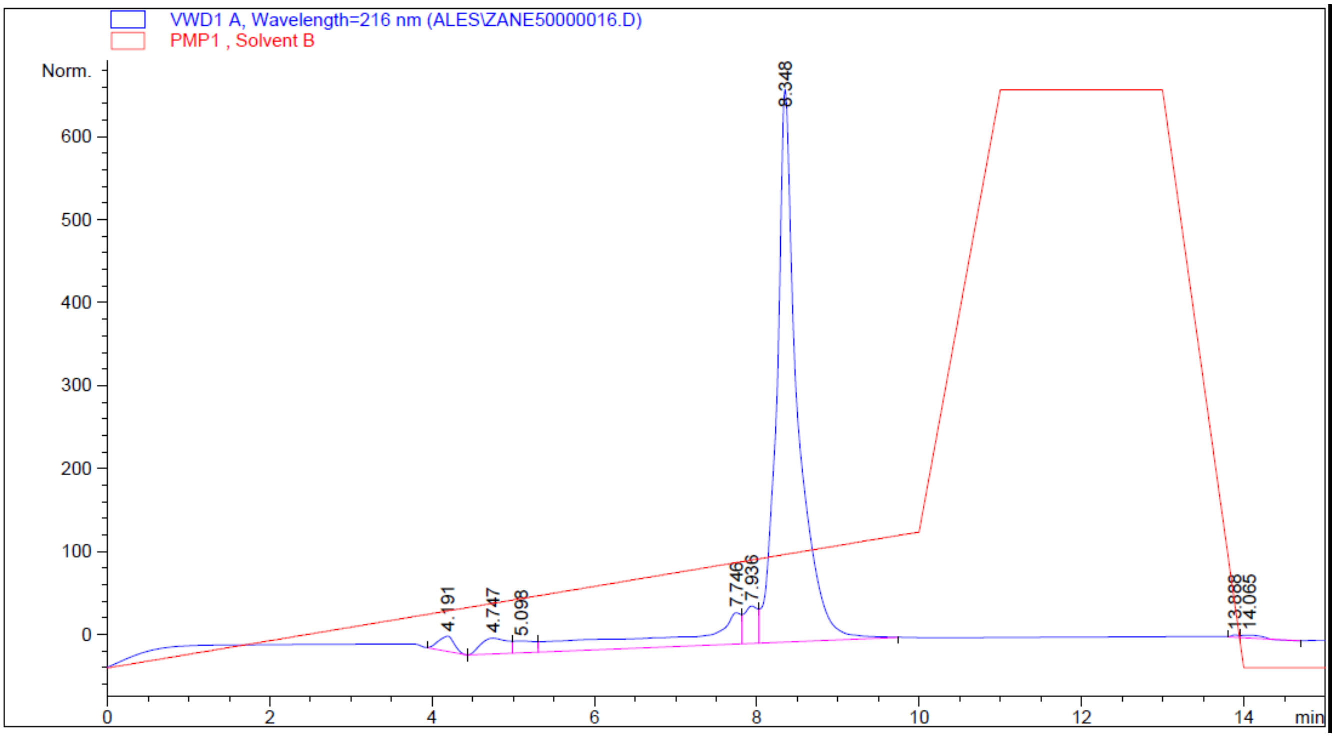

Figure S21: HPLC profile of peptide **6** after 1 hour in the presence of pronase.

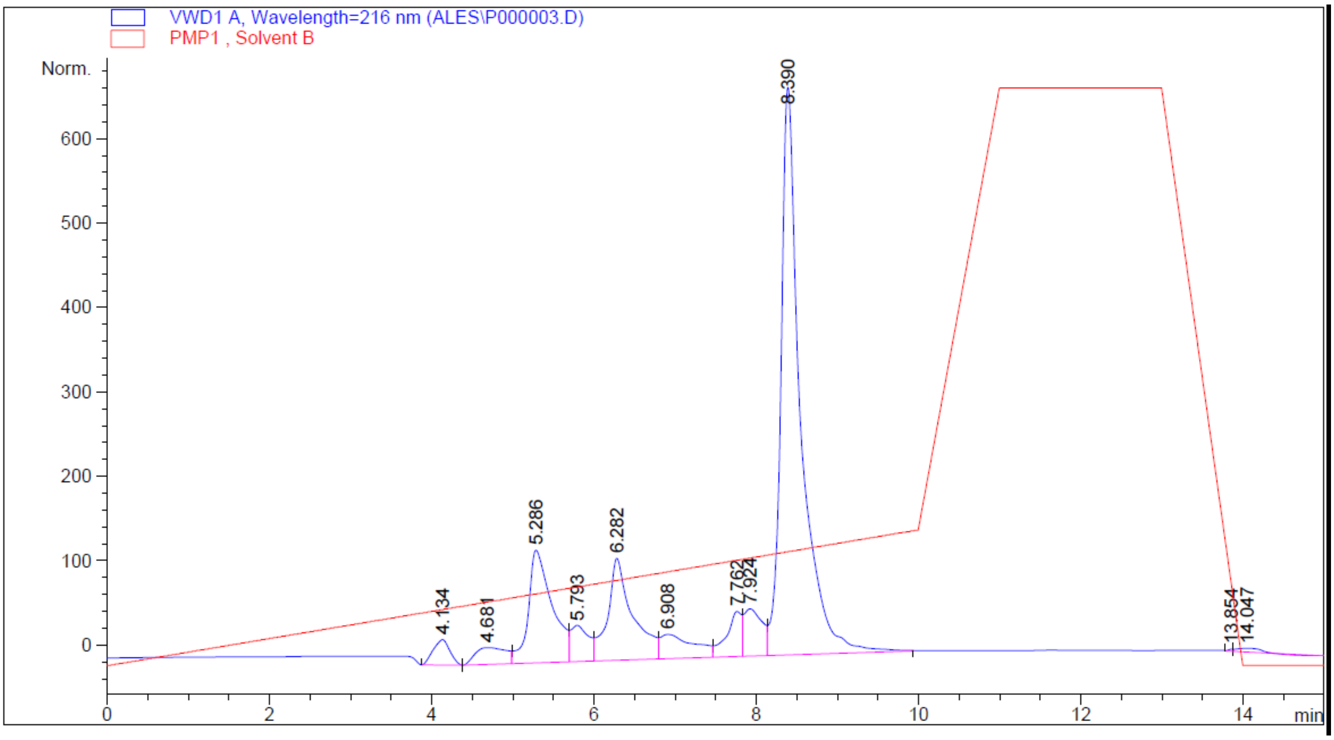

Figure S22: HPLC profile of peptide **6** after 6 days in the presence of pronase.

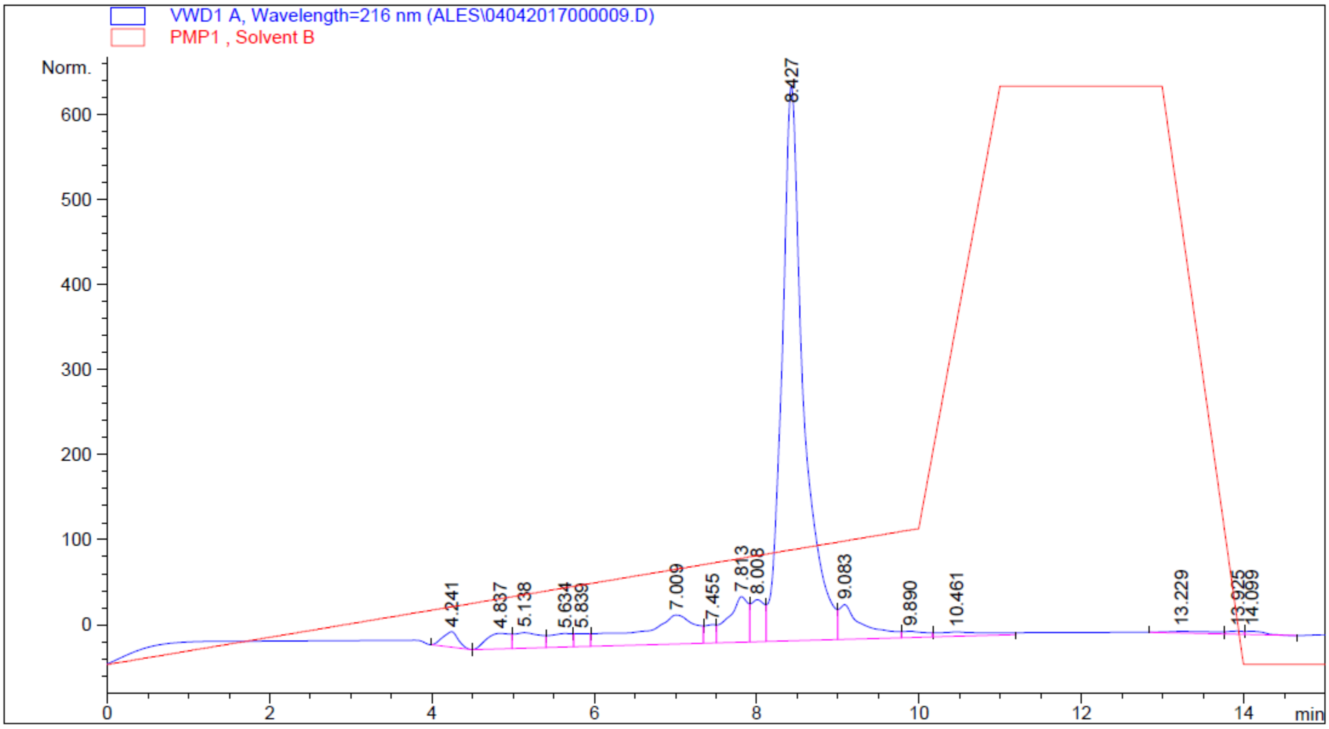

Figure S23: HPLC profile of peptide **6** after 6 days in trypsin.

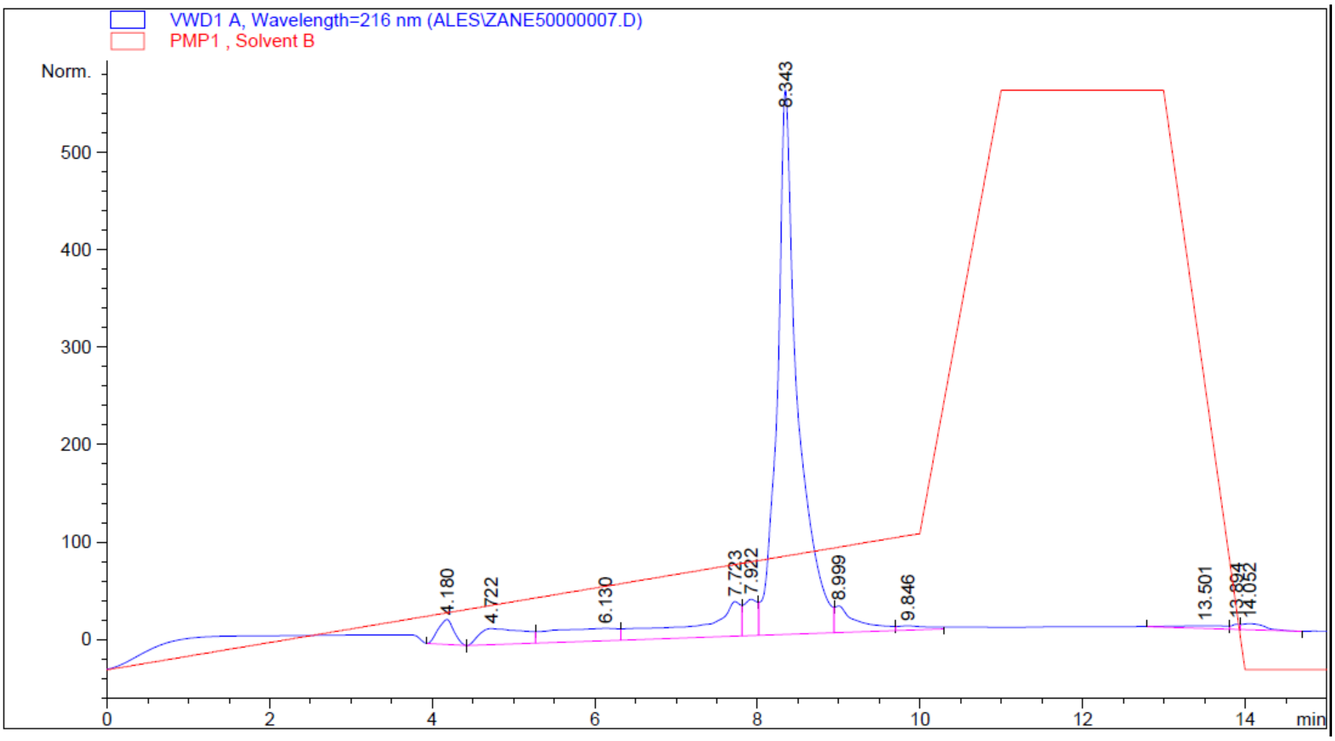

Figure S24: HPLC profile of peptide **6** after 75 min in chymotrypsin.
